# Dictionary learning in Fourier-transform scanning tunneling spectroscopy

**DOI:** 10.1038/s41467-020-14633-1

**Published:** 2020-02-26

**Authors:** Sky C. Cheung, John Y. Shin, Yenson Lau, Zhengyu Chen, Ju Sun, Yuqian Zhang, Marvin A. Müller, Ilya M. Eremin, John N. Wright, Abhay N. Pasupathy

**Affiliations:** 1 Department of Physics, Columbia University, New York, NY 10027 USA; 2 Department of Electrical Engineering, Columbia University, New York, NY 10027 USA; 3 Institut für Theoretische Physik III, Ruhr-Universität Bochum, 44801 Bochum, Germany; 4 National University of Science and Technology MISiS, 119049 Moscow, Russian Federation

**Keywords:** Image processing, Imaging techniques, Electronic properties and materials, Superconducting properties and materials, Imaging techniques

## Abstract

Modern high-resolution microscopes are commonly used to study specimens that have dense and aperiodic spatial structure. Extracting meaningful information from images obtained from such microscopes remains a formidable challenge. Fourier analysis is commonly used to analyze the structure of such images. However, the Fourier transform fundamentally suffers from severe phase noise when applied to aperiodic images. Here, we report the development of an algorithm based on nonconvex optimization that directly uncovers the fundamental motifs present in a real-space image. Apart from being quantitatively superior to traditional Fourier analysis, we show that this algorithm also uncovers phase sensitive information about the underlying motif structure. We demonstrate its usefulness by studying scanning tunneling microscopy images of a Co-doped iron arsenide superconductor and prove that the application of the algorithm allows for the complete recovery of quasiparticle interference in this material.

## Abstract (web summary)

Aperiodic structure imaging suffers limitations when utilizing Fourier analysis. The authors report an algorithm that quantitatively overcomes these limitations based on nonconvex optimization, demonstrated by studying aperiodic structures via the phase sensitive interference in STM images.

## Introduction

The past few decades have seen dramatic advances in the understanding of the structure of materials via scattering and microscopy techniques. Scattering techniques are useful when perfect periodicity exists in a material, while microscopy is well suited for specimens that lack periodicity. Recent advances in microscopy techniques, when coupled with improved computing power, have enabled the scientific community to generate massive, multi-dimensional spatial images of specimens as a function of control parameters, such as time, energy, and applied stimulus. Examples of such advanced tools include super-resolution optical microscopy to inspect the structure of proteins beyond the diffraction limit^1^, scanning transmission electron microscopy to examine the chemical structure of materials at the atomic scale^2^, and scanning tunneling microscopy (STM) to visualize the quantum electronic structure of surfaces with atomic resolution^3^. Fundamentally, a microscope image represents the interaction between the probe and the specimen, and often times sophisticated analysis must be performed to uncover the scientific content present in the image. Specimens of interest for STM studies include metals^4^, two-dimensional (2-D) materials^5^, unconventional superconductors^6^, topological materials^7,8^, and charge^9^ and spin^10,11^ ordered materials, among others. Image analysis of these materials has provided several unique insights into the quantum electronic structure and interactions present within them. Many microscopy techniques utilize the Fourier transform (FT)^12,13^ for analysis, revealing the characteristic wavelengths present in the image, which are then related to a scientific theory of the specimen being studied. When perfect periodicity exists in an image, the FT provides a concise and accurate description of the image^6,7,9,14,14–18^. However, when applied to aperiodic images, the FT suffers from phase noise leading to a fundamental loss of information^19,20^. With the proliferation of new computing techniques, one may wonder if the maturation of optimization algorithms can be leveraged to extract more information from a microscopy image than through the FT.

In this work, we consider a class of images that are of particular importance to microscopy—those that can be perceived as a basic motif, called a kernel, that is repeated aperiodically across the image. Examples of kernels include electronic scattering patterns around atomic defects (in STM) and fluorescence from individual proteins (in optical microscopy). We present the development of an algorithm, based on nonconvex optimization, for analyzing such images that quantitatively extracts the principal motifs present in an image. We demonstrate that this algorithm can elucidate fundamentally new information unavailable through traditional FT analysis. While our methods are generally applicable to a wide range of microscopy techniques, in this work we focus on its application to STM.

## Results

### STM and scanning tunneling spectroscopy

**Fig. 1:**
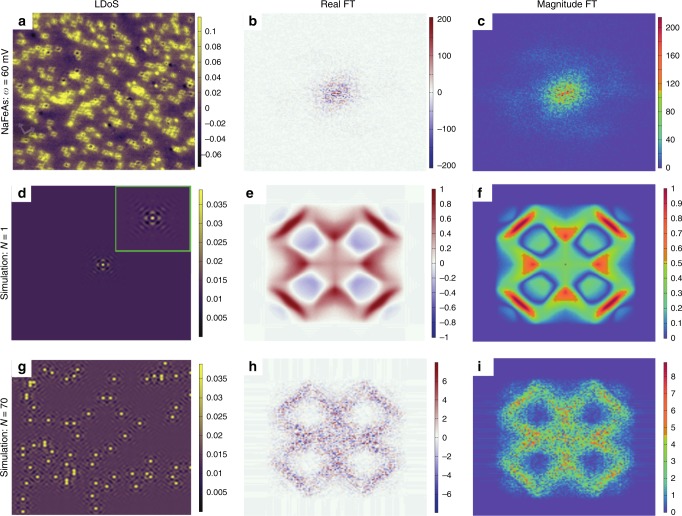
Shortcomings of the Fourier transform analysis. **a** The experimentally obtained LDoS of NaFeAs over a 100 × 100 nm^2^ area of the sample at energy *ω* = 60 mV from ref. ^18^ with junction conditions *V* = − 100 mV and *I* = 300 pA measured at *T* = 26 K. Color bar units are nS. **b**, **c** Real part and magnitude, respectively, of the FT of **a** showing large phase noise. Color bar units are nS^−1^. **d** Simulated image of scattering from a single point defect. **e**, **f** Real part and magnitude, respectively, of the FT of **d**. The LDoS scale in **d** was chosen such that the FT magnitude for the simulated single defect had a maximum of 1. **g**–**i** Corresponding simulated LDoS and FTs for scattering from 70 point defects that are randomly distributed in space. The color bars for **d**–**i** are in arbitrary units. All FT spectra are shown for  −3*π*/5*a* ≤ *q*_*x*_, *q*_*y* _≤ 3*π*/5*a*. Comparison with the single defect case shows that noise in the FTs arises from the random placement of defects. Furthermore, the maximum amplitude of the FT scales approximately as $\sqrt{N}$, resulting in a loss of signal fidelity in the FT analysis.

STM and scanning tunneling spectroscopy produces 2-D spectroscopic maps of the local density of states (LDoS) at position **x** with energy *ω*, forming a three-dimensional dataset. The contrast in these images stems from local spatial variations of the LDoS, denoted as *δρ*(**x**, *ω*). Measurements in which *δρ*(**x**, *ω*) is ascribed to material defects that cause electron scattering and interference^4^ are particularly interesting, and such maps are often called quasiparticle interference (QPI) maps. Analysis of QPI maps has uncovered information on the dispersion relations and scattering processes in semimetals^21,22^, high-temperature superconductors^6,14,18,23^, and other systems. Let us suppose that the LDoS pattern created by a single defect located at **x** with energy *ω* is *δρ*_0_(**x**, *ω*), and that the STM image is composed from *N* defects located at **x**_1_, **x**_2_, …, **x**_*N*_:

1 $$\delta \rho ({\bf{x}},\omega)=\mathop {\sum}\limits_{j=1}^{N}{c}_{j}\delta {\rho }_{0}({\bf{x}}-{{\bf{x}}}_{j},\omega),$$

where *c*_*j*_ are constants. A real-world example of such an image is shown in Fig. 1a, obtained on the pnictide superconductor NaFeAs^18^.

According to scattering theory, the FT of the QPI image of an individual defect, *δρ*_0_(**q**, *ω*) = ∫d**x** *e*^−*i***q**⋅**x**^*δρ*_0_(**x**, *ω*), is correlated with the underlying electronic structure of the material^14–16^. Many quantum materials previously studied by STM, examples of which include superconducting cuprates^6^, pnictides^18^ and chalcogenides^10^, charge density wave materials^9^, topological insulators^7^, and correlated oxides^24^ have sufficient disorder so that the LDoS signatures of different defects overlap. In this situation, it is not possible to identify the isolated defect signature through inspection. Instead, the traditional analysis^9^ proceeds by taking the FT of the entire STM image *δρ*(**x**, *ω*) in (1):

2 $$\delta \rho ({\bf{q}},\omega)=\int {\rm{d}}{\bf{x}}\ {e}^{-i{\bf{q}}\cdot {\bf{x}}}\delta \rho ({\bf{x}},\omega)=\delta {\rho }_{0}({\bf{q}},\omega)\mathop {\sum}\limits_{j=1}^{N}{c}_{j}\exp \{-i{\bf{q}}\cdot {{\bf{x}}}_{j}\}.$$

While the quantity of interest for QPI analysis is *δρ*_0_(**q**, *ω*), the experimental FT image contains a frequency-varying, complex-valued phase factor, ${\mathcal{P}}({\bf{q}})\equiv {\sum }_{j=1}^{N}{c}_{j}\exp \{-i{\bf{q}}\cdot {{\bf{x}}}_{j}\}$. This is illustrated in Fig. 1b, where the real part of the FT (Re-FT) displays wild oscillations due to ${\mathcal{P}}({\bf{q}})$. To mitigate this, the magnitude of the FT (mag-FT) is taken, and the analysis proceeds by assuming that ${\mathcal{P}}({\bf{q}})$ is approximately constant in magnitude so that $\left|{\mathcal{P}}({\bf{q}})\right|\approx \bar{c}\sqrt{N}$, where $\bar{c}$ is the average value of the *c*_*j*_. The result of this procedure is illustrated in Fig. 1c, showing that debilitating noise still persists in the FT after taking the modulus. Moreover, the procedure of obtaining the mag-FT effectively eliminates half of the useful information in the complex-valued FT, annihilating all of the phase information from electron scattering processes originally present in real space. Intense peaks and contours in the real and imaginary parts of *δρ*(**q**, *ω*) are experimental indicators of dominant scattering wavevectors and order parameter symmetries, which can reveal important properties about the superconducting gap function sign structure^25,26^ and surface states of topological insulators^27,28^. However, random phase noise fluctuations in experimental QPI spectra make comparisons with theoretical QPI calculations difficult^29^.

**Fig. 2:**
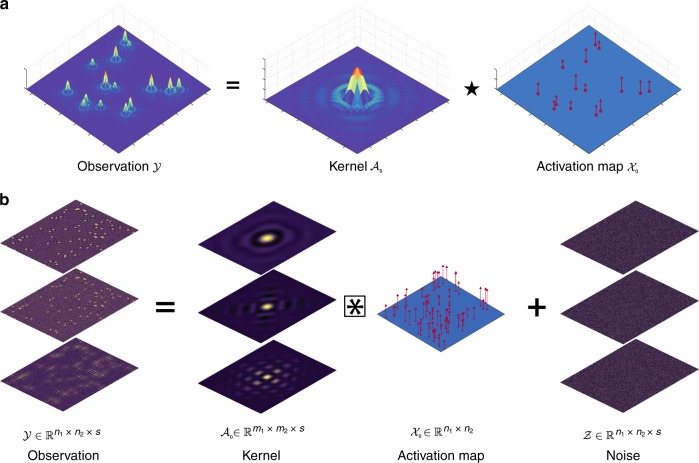
Convolutional data model for STM. **a** Schematic representation of the convolutional data model ${\mathcal{Y}}={{\mathcal{A}}}_{0}\star {{\mathcal{X}}}_{0}$ for a noise-free simulated STM measurement. **b** Schematic representation of STM analysis as a high-dimensional deconvolution problem. Each constant-energy plane of ${{\mathcal{A}}}_{0}$ is convolved with the known activation map ${{\mathcal{X}}}_{0}$ and combined with additive noise ${\mathcal{Z}}$ to produce the noisy observation ${\mathcal{Y}}$. Algorithms for SBD seek to efficiently estimate ${{\mathcal{A}}}_{0}$ and ${{\mathcal{X}}}_{0}$ given the noisy measurement ${\mathcal{Y}}$.

An improved analysis technique to FT-STM would identify the location and the LDoS signature associated with each defect in a quantitatively rigorous fashion that respects experimental and material-specific constraints. For instance, defects remain fixed in position across a series of STM images in which the measurement bias voltage is varied (see Fig. 2). In this work, we present an analysis technique based on nonconvex optimization that possesses these desirable features while being broadly applicable to other forms of microscopy and image analysis.

### Connection with sparse blind deconvolution

Our algorithm is based on a deconvolutional procedure illustrated in Fig. 2. The image (denoted as ${\mathcal{Y}}$) in Fig. 2a was produced by simulating the effect of quasiparticle scattering from numerous point defects randomly distributed across the image. At a given bias voltage, the image consists of a recurrent scattering pattern (called the kernel ${{\mathcal{A}}}_{0}$) convolved with the locations and relative weights of each defect (called the activation map ${{\mathcal{X}}}_{0}$) as illustrated in Fig. 2a and represented as ${\mathcal{Y}}={{\mathcal{A}}}_{0}\star {{\mathcal{X}}}_{0}$. The underlying challenge in our analysis is to invert the procedure—starting with an STM image, determine the kernel and its corresponding activation map. The kernel and activation map are easily identified by inspection in Fig. 2a; however, this becomes a highly non-trivial problem in the presence of many overlapping kernels and experimental noise. Similar convolutional models are used in neuroscience to model neuron spike patterns^19^ and in systems biology to capture responses of the endocrine system^20^. In contrast, our algorithm focuses on a 2-D signal.

When ${{\mathcal{A}}}_{0}$ contains multiple slices, with each slice corresponding to a different bias voltage, we mathematically express the proposed model for STM measurements by collecting the convolutions for each voltage slice using the notation

3 $${\mathcal{Y}}={{\mathcal{A}}}_{0} \otimes {{\mathcal{X}}}_{0}+{\mathcal{Z}},$$

which is schematically depicted in Fig. 2b. The activation map ${{\mathcal{X}}}_{0}$ is shared globally across all measurement biases and ${\mathcal{Z}}$ is an additive noise tensor. The task of recovering both ${{\mathcal{A}}}_{0}$ and ${{\mathcal{X}}}_{0}$ given ${\mathcal{Y}}$ is known as the sparse blind deconvolution (SBD) problem.

### Formulating the SBD problem

Over the past decade, a wealth of heuristics and applications for sparse signal recovery have been developed, often leading to efficient algorithms in theory as well as in practice^30,31^ (see Supplementary Note 2). We investigate the following heuristic for producing estimates $\hat{{\mathcal{A}}}$ and $\hat{{\mathcal{X}}}$ for ${{\mathcal{A}}}_{0}$ and ${{\mathcal{X}}}_{0}$, by posing an optimization problem based on (3):

4 $$\hat{{\mathcal{A}}}=\arg \ \min _{{\mathcal{A}}} \min _{{\mathcal{X}}}\left[{\psi }_{\lambda }\left({\mathcal{A}},{\mathcal{X}}\right)\equiv \frac{1}{2}{\left\Vert {\mathcal{A}} \otimes {\mathcal{X}}-{\mathcal{Y}}\right\Vert }_{\mathrm{F}}^{2}+\lambda r({\mathcal{X}}),\right]$$

which allows one to recover $\hat{{\mathcal{X}}}=\arg \ {\min }_{{\mathcal{X}}}{\psi }_{\lambda }(\hat{{\mathcal{A}}},{\mathcal{X}})$.

This is similar to prem .vious formulations proposed for various SBD applications: the Frobenius norm term ${\left\Vert \cdot \right\Vert }_{\mathrm{F}}^{2}$ promotes data fidelity upon minimization $({\mathcal{Y}}\simeq \hat{{\mathcal{A}}} \otimes \hat{{\mathcal{X}}})$, and a regularization term $r(\hat{{\mathcal{X}}})$ is chosen, such as the *ℓ*_1_ norm, so that the minimization encourages $\hat{{\mathcal{X}}}$ to be sparse, with *λ* ≥ 0 governing the trade-off between the two objectives. However, most SBD applications focus on signal enhancement that uses the convolutional model as a rough guideline, leading to a weak notion of accurate estimation^32^. In contrast, the convolutional model fits naturally into the STM setting, in which robust, consistent results are paramount for scientific investigation. These considerations prompt a number of choices that are not emphasized in previous heuristics, such as the domain of ${\mathcal{A}}$, form of $r(\hat{{\mathcal{X}}})$, and refinement of the estimates.

In order to solve this optimization problem, we present the SBD-STM algorithm:

**Algorithm 1** Complete SBD-STM procedure

**Input:**

- Observation ${\mathcal{Y}}\in {{\mathbb{R}}}^{{n}_{1}\times {n}_{2}\times s}$, kernel size $\left({m}_{1},{m}_{2}\right)$, initial *λ*_0_ ≥ 0, decay rate $\alpha \in \left[0,1\right)$, and final *λ*_end_ ≥ 0.

**Initial phase:**

1. Randomly initialize: $${{\mathcal{A}}}^{\left(0\right)}\in {\mathcal{S}}={{\mathbb{S}}}^{{m}_{1}\times {m}_{2}\times s}$$.
2. $${{\mathcal{A}}}_{* }^{\left(0\right)},{{\mathcal{X}}}_{* }^{\left(0\right)}\leftarrow$$ASolve$$({{\mathcal{A}}}^{(0)},{\lambda }_{0},{\mathcal{Y}})$$.

**Refinement phase:**

1. Lifting: Get ${{\mathcal{A}}}^{\left(1\right)}\in {S}^{\prime}={{\mathbb{S}}}^{{m}_{1}^{\prime}\times {m}_{2}^{\prime}\times s}$ by zero-padding the edges of ${{\mathcal{A}}}_{* }^{\left(0\right)}$ with a border of width $\left\lfloor \frac{{m}_{i}}{2}\right\rfloor$.
2. Set *λ*_1_ = *λ*_0_.
3. Continuation: **Repeat** for *k* = 1, 2, …  **until** *λ*_*k*_ ≤ *λ*_end_,
  - $${{\mathcal{A}}}_{* }^{\left(k\right)},{{\mathcal{X}}}_{* }^{\left(k\right)}\leftarrow$$ASolve$$\left({{\mathcal{A}}}^{\left(k\right)},{\lambda }_{k},{\mathcal{Y}},{{\mathcal{X}}}_{* }^{\left(k-1\right)}\right)$$,
  - Centering:
    i. Find the size *m*_1_ × *m*_2_ submatrix of ${{\mathcal{A}}}_{* }^{\left(k\right)}$ that maximizes the Frobenius (square) norm across all *m*_1_ × *m*_2_ submatrices.
    ii. Get ${{\mathcal{A}}}^{\left(k+1\right)}$ by shifting ${{\mathcal{A}}}_{* }^{\left(k\right)}$ so that the chosen *m*_1_ × *m*_2_ restriction is in the center, removing and zeropadding entries as needed.
    iii. Normalize ${{\mathcal{A}}}^{\left(k+1\right)}$ so it lies in ${{\mathcal{S}}}^{\prime}$.
    iv. Shift ${{\mathcal{X}}}_{* }^{\left(k\right)}$ along the anti-parallel vector to the shift of ${{\mathcal{A}}}_{* }^{\left(k\right)}$.
  - Set *λ*_*k*+1_ = *αλ*_*k*_.

**Output:**

1. Extract $\hat{{\mathcal{A}}}\in {\mathcal{S}}$ by extracting the restriction of the final ${{\mathcal{A}}}^{\left(k+1\right)}$ to the center *m*_1_ × *m*_2_ window.
2. Find the corresponding activation map $\hat{{\mathcal{X}}}\in {{\mathbb{R}}}^{{n}_{1}\times {n}_{2}}$ by solving ${\min }_{{\mathcal{X}}}{\psi }_{{\lambda }_{k}}(\hat{{\mathcal{A}}},{\mathcal{X}})$.

**Function** Asolve

**Input:**

- Current kernel, ${{\mathcal{A}}}_{{\rm{in}}}$, current sparsity parameter, *λ*_in_, the observation ${\mathcal{Y}}$, current activation map ${{\mathcal{X}}}_{{\rm{in}}}$ (Refinement Phase)

**Minimization**

**if** Initial Phase **then**

1. Initialize ${\mathcal{X}}$ as a zero matrix of size (*n*_1_, *n*_2_)
2. ${{\mathcal{X}}}_{1}\leftarrow$ Minimize ${\mathcal{X}}$ for ${\psi }_{{\lambda }_{{\rm{in}}}}({{\mathcal{A}}}_{{\rm{in}}},{\mathcal{X}})$ using the FISTA algorithm^33^.

 **else**

 $${{\mathcal{X}}}_{1}\leftarrow {{\mathcal{X}}}_{{\rm{in}}}$$

**end** **if**

1. ${{\mathcal{A}}}_{{\rm{out}}}\leftarrow$ Minimize ${\mathcal{A}}$ for ${\psi }_{{\lambda }_{{\rm{in}}}}({\mathcal{A}},{{\mathcal{X}}}_{1})$ using the Riemannian Trust-Region Method (RTRM) over the sphere^34^.
2. ${{\mathcal{X}}}_{{\rm{out}}}\leftarrow$ Minimize ${\mathcal{X}}$ for ${\psi }_{{\lambda }_{{\rm{in}}}}({{\mathcal{A}}}_{{\rm{out}}},{\mathcal{X}})$ using FISTA.

**Output:**

- $${{\mathcal{A}}}_{{\rm{out}}}$$, $${{\mathcal{X}}}_{{\rm{out}}}$$.

See Supplementary Notes 2 and 3 for further discussion on formulating and solving Eq. (4), and Supplementary Note 4 for how our approach to SBD can be applied to image deblurring by using an objective similar to Eq. (4).

### The blind deconvolution approach

**Fig. 3:**
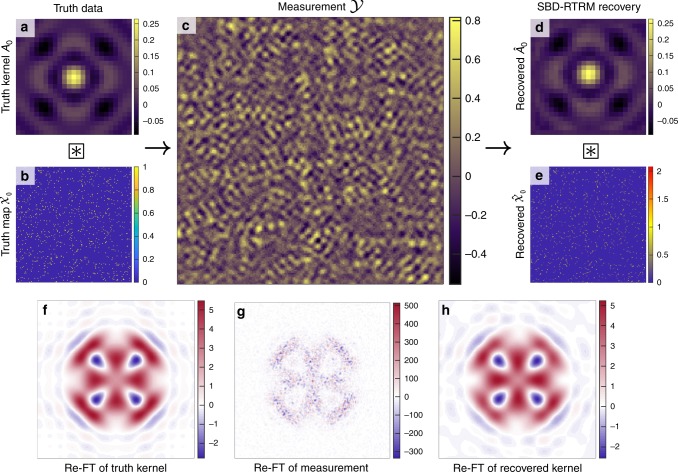
Illustrating the SBD-STM procedure. The simulated kernel (size 25 × 25 pixels) in **a** is convolved with the activation map in **b** and added to random noise with SNR ≈0.792 to produce the simulated noisy measurement shown in **c**. The simulated measurement is a 185 × 185 pixel image with a defect concentration of *θ* = 2.73%. **d** Recovered kernel and **e** recovered activation map obtained using SBD-STM with *λ* = 0.1, showing excellent agreement with simulation inputs in **a**, **b**. The Re-FTs of the truth kernel, noisy measurement, and the recovered kernel from SBD-STM, shown in **f**–**h**, respectively. The Re-FT spectra are all shown with  − 3*π/*5*a* ≤ *q*_*x*_, *q*_*y*_ ≤ 3*π/*5*a*, and all color bars are in arbitrary units. The SBD-STM-based FT shows both vastly improved SNR and a phase-sensitive recovery of the scattering interference.

To demonstrate the strength of SBD-STM, consider the situation illustrated in Fig. 3. We generated a simulated observation ${\mathcal{Y}}$ using a ground truth scattering pattern ${{\mathcal{A}}}_{0}$ similar to Fig. 1d and a dense, randomly generated activation map ${{\mathcal{X}}}_{0}$ shown in Fig. 3b. Convolving the truth data with the activation map and adding significant white noise with variance *η* so that the signal-to-noise ratio (SNR) is less than unity, with ${\rm{SNR}}\equiv \frac{{\mathrm{var}}({{\mathcal{A}}}_{0})}{\eta }$, we generate the image shown in Fig. 3c. With many overlapping kernels and substantial noise, it is a futile task to accurately identify the underlying kernel and activation map of the image through visual inspection. However, as shown in Fig. 3d, e, SBD-STM successfully recovers a kernel $\hat{{\mathcal{A}}}$ and its associated activation map $\hat{{\mathcal{X}}}$ that closely resemble the truth data. The results shown in Fig. 3 were obtained with a fixed *λ* = 0.1. The scaling of the activation map entries is due to the choice of *λ*, and the noise also introduces blurring in the activation map^35^. Despite this, the overall features of the activation map and the recovered kernel are remarkably similar to the ground truth.

In Fourier space, the Re-FT of ${\mathcal{Y}}$ is missing crucial features of the true Re-FT spectrum and has noise fluctuations  ≈100 times that of the true transform. However, the Re-FT of the SBD-STM recovered kernel is consistent with the true Re-FT in both its structure and amplitude.

In our implementation, SBD-STM yields an activation map shared across all bias voltages. This not only reveals the spatial distribution of the defect kernels but also naturally improves the accuracy of the SBD-STM recovered kernels at bias energies with noisy measurements. Consequently, SBD-STM returns more physically meaningful results when data from multiple biases are simultaneously analyzed than if each constant-bias slice of ${\mathcal{Y}}$ were individually analyzed. SBD-STM results on a simulated noisy STM dataset with 41 bias voltages are found in Supplementary Note 5, demonstrating that SBD-STM is successful in optimizing the objective function with STM constraints in mind.

### Performance characterization

**Fig. 4:**
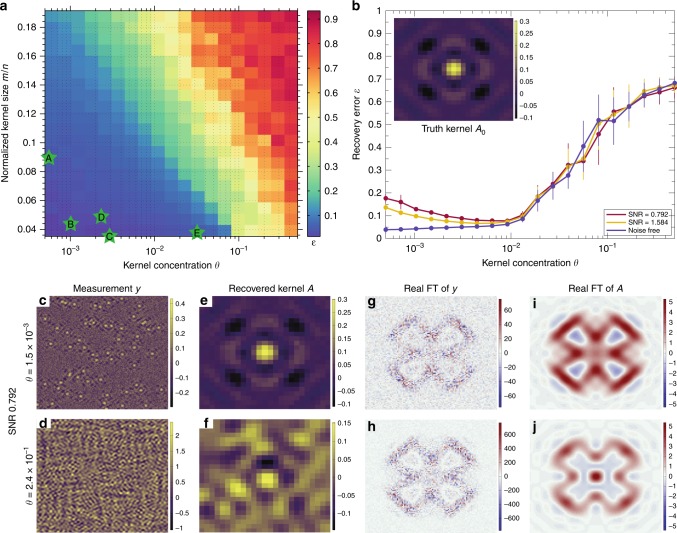
Benchmarking SBD-STM performance. **a** SBD-STM performance phase diagram from noise-free simulated results on images of area *n* = 256 × 256, plotting the error metric $\epsilon ({\hat{{\mathcal{A}}}}_{\theta ,\eta },{{\mathcal{A}}}_{0})$ as a function of normalized kernel size and concentration *θ* (the number of defects scaled by the number of pixels). Each point on the diagram is determined from the mean of 20 independent simulated measurements. SBD-STM performs well in the blue regions, but begins to fail in the red regions, at very high kernel size or concentration. Green stars labeled A, B, C, D, and E indicate representative images from refs. ^5,7,9,18,48^, respectively. **b** Performance of SBD-STM in the presence of additive noise in the measurement at a fixed value of the normalized kernel size *m*∕*n* = 0.14. Error bars represent the standard deviation of the error metric from the 20 simulated measurements at each Kernel concentration. The inset shows the truth kernel used in the simulations. **c**–**f** Examples of simulated measurements from **b** and their corresponding SBD-STM recovered kernels. **g**–**j** A comparison of the Re-FTs of the measurement and the SBD-STM recovered kernel, indicating that SBD-STM consistently outperforms FT-STM across all parameter ranges. All Re-FT spectra are shown with  − 3*π/*5*a* ≤ *q*_*x*_, *q*_*y*_ ≤ 3*π/*5*a*, and color bars for **c**–**j** are in arbitrary units.

Before invoking SBD-STM on experimental data, we must understand its limitations and domain of applicability. The complexity of the STM deconvolution problem varies depending on the SNR and the overlap tendency of nearby defects. A series of numerical experiments on simulated data were performed to investigate the effects of defect concentration *θ*—the probability that any entry of ${{\mathcal{X}}}_{0}$ is truly non-zero—and additive measurement noise on the expected success of SBD-STM. Simulated STM images are produced in a similar fashion as in Fig. 3, and the performance of SBD-STM was assessed as a function of four adjustable parameters—the image size *n* ≡ *n*_1_ × *n*_2_, kernel size *m* ≡ *m*_1_ × *m*_2_, kernel concentration *θ*, and SNR. Details of the data generation and simulation work are contained in Supplementary Notes 1 and 6. To assess the accuracy of kernel recovery in real space, we define the real-space recovery error metric as $\epsilon ({\hat{{\mathcal{A}}}}_{\theta ,\eta },{{\mathcal{A}}}_{0})\equiv \frac{2}{\pi }\arccos |\langle {\hat{{\mathcal{A}}}}_{\theta ,\eta },{{\mathcal{A}}}_{0}\rangle|$, with $\langle {\hat{{\mathcal{A}}}}_{\theta ,\eta },{{\mathcal{A}}}_{0}\rangle$ denoting the inner product of the vectorizations of ${\hat{{\mathcal{A}}}}_{\theta ,\eta }$ and ${{\mathcal{A}}}_{0}$, which are the recovered and truth kernels, respectively. Figure 4a depicts a normalized defect size $\frac{m}{n}$ vs. concentration *θ* phase diagram to explore the interplay between $\frac{m}{n}$ and *θ* on real-space algorithmic accuracy $\epsilon ({\hat{{\mathcal{A}}}}_{\theta },{{\mathcal{A}}}_{0})$ in noise-free (*η* = 0) simulated measurements. We observe a phase transition in SBD-STM performance in the $\frac{m}{n}-\theta$ plane. The bottom left of the plot captures situations in which the defects have negligible probability of overlapping, facilitating the near-perfect deconvolution of the noise-free image. Increasing either $\frac{m}{n}$ or *θ* introduces error in the kernel recovery due to increased overlapping between defects. Practically, $\frac{m}{n}$ can be reduced by increasing the overall STM measurement area *n* in an attempt to perform deconvolution-by-inspection. However, at high defect concentrations *θ* or moderate noise levels, this strategy cannot guarantee success, while SBD-STM can still return reliable estimates.

Next, we briefly discuss the SBD-STM performance as a function of noise in the signal. Figure 4b shows the evolution of $\epsilon ({\hat{{\mathcal{A}}}}_{\theta ,\eta },{{\mathcal{A}}}_{0})$ as a function of defect concentration *θ* for three values of SNR ranging from noise free to noise dominated (SNR = 0.792). At high noise levels, performance error fluctuations in the far left of the plot appear because of the statistically futile challenge of accurately identifying low-density, low-intensity motifs under high levels of noise. The error curves appear to converge into a narrow band when *θ* ≳ 0.01, demonstrating that the algorithm is robust to a wide range of SNRs for higher concentrations. By *θ* ≈ 0.2, the defect concentrations are sufficiently dense so that virtually all defect kernels are overlapping, causing SBD-STM to collapse and return unreliable estimates. These trends persist when the kernel size and SNR are further increased, as described in Supplementary Note 6. Altogether, our simulations show that in a wide range of parameters (Fig. 4a), SBD-STM performs splendidly (*ϵ* < 0.1) and consistently outperforms the usual FT-STM methodology even in the presence of considerable noise, as seen in Fig. 4c–j.

### Application to real data

**Fig. 5:**
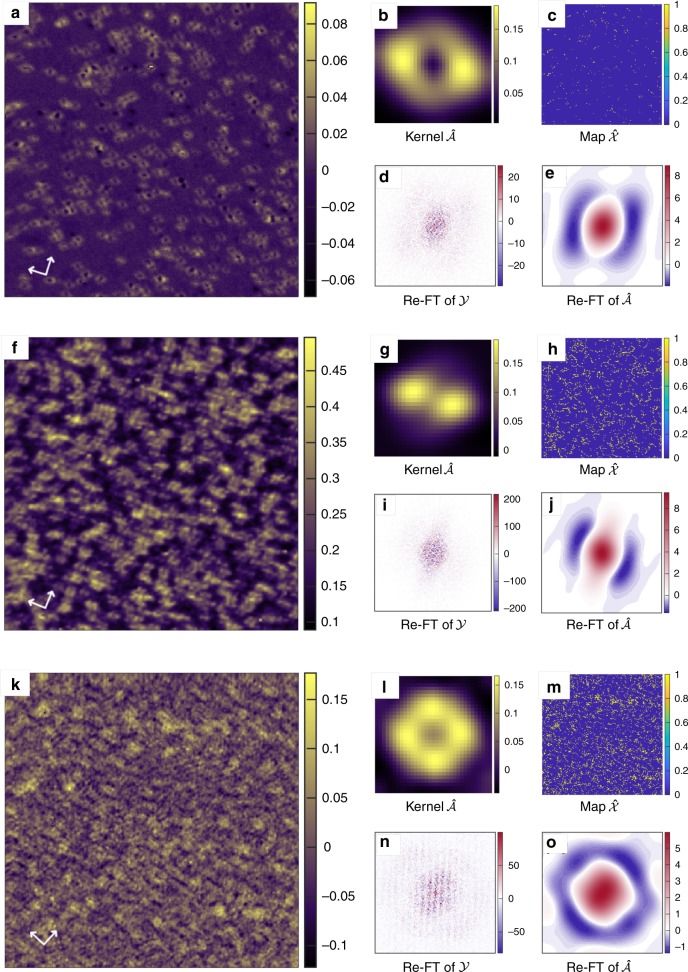
Application to real data. SBD-STM recoveries with increasing density of defects. In the left column, we have conductance maps with Co defects in NaFe_1−*x*_Co_*x*_As of **a** high-quality (*T* = 71 K, *I* = 59 pA, *V*_set_ = −100 mV, 114 × 114 nm^2^), **f** *x* = 0.015 (*T* = 6 K, *I* = −200 pA, *V*_set_ = −20 mV, 50.4 × 50.4 nm^2^), and **k** *x* = 0.02 (*T* = 5.9 K, *I* = 200 pA, *V*_set_ = −50 mV, 250 × 250 nm^2^) biased at energies of **a** 40 meV, **f** 5 meV, and **k**  − 7 meV. The arrows indicate the principal directions of the lattice. In the right column, clockwise from the top left, we have the recovered kernel **b**, **g**, **l**, the recovered activation map **c**, **h**, **m**, the Re-FT of the kernel **d**, **i**, **n**, and the Re-FT of the conductance map **e**, **j**, **o**, for comparison. The recovered kernels have been given 2-fold rotational symmetry, and all activations have been set to 1 to make them more readily visible. The STM spectroscopy maps have been drift corrected as outlined in Supplementary Note 7. Color bars for real-space images are in units of nS, while color bars for Fourier images are in units of nS^−1^.

The results obtained above on synthetic data show that SBD-STM is able to recover kernels even at high defect density and in the presence of significant white noise, where alternate techniques such as manual detection fail. However, one might still question whether the method will work on real STM data, which might have other types of noise or errors, which we have not accounted for in our synthetic data. In order to investigate this fully, we now apply SBD-STM to investigate a set of experimentally obtained STM images of NaFe_1−*x*_Co_*x*_As at different values of *x* = 0.0, 0.015 and 0.02. At *x* = 0.0 (parent compound), no additional cobalt dopants are present in the lattice, and the only defects present are those intrinsic to the crystal, which are at a concentration of about 1%. This compound has been previously studied via STM and the data have been analyzed using the standard FT technique^18^. To contrast with the standard FT-STM analysis, we implement SBD-STM on raw experimental data to demonstrate the significant improvement in data fidelity of the Re-FT. Shown in Fig. 5b, c are the recovered Kernel and activation map, respectively, from the map shown in Fig. 5a. Figure 5d, e show the Re-FT of the entire image and of the kernel, respectively, over the same range in Fourier space. The phase sensitive recovery of the FT in Fig. 5e when compared to Fig. 5d is immediately apparent. We note that for this particular doping the individual kernels are well separated, and the kernel can be isolated directly by eye from the large area image in Fig. 5a. We show the application of SBD-STM to this sample to illustrate that the recovered kernel is indeed what one would expect by direct measurement around an individual defect.

We now turn to the sample with *x* = 0.015, a STS image of which is shown in Fig. 5f. At this doping, we see that there are regions of high-density clustering of the kernels, where the clustering is dense enough such that the individual kernels overlap and become difficult to resolve. In Fig. 5g, h, we show the corresponding recovery for the kernel and activation map, respectively. The Re-FT of the entire map and the recovered kernel are shown in Fig. 5i, j, respectively. At this defect level, we can very occasionally see isolated defects, and the recovered kernel is seen to nicely match with the differential conductance around these isolated defects.

Finally, we consider a sample that is optimally doped (highest *T*_c_) with a nominal cobalt concentration of *x* = 0.02. Shown in Fig. 5k is an STS image obtained on this sample at *T* = 5.9 K. At this doping level, there are no isolated impurities present anywhere in the sample, and the kernel cannot be manually recovered. The SBD-STM is able to recover the kernel and activation map as shown in Fig. 5l, m respectively. The Re-FT of the entire image and of the recovered kernel are shown in Fig. 5n, o, respectively. We note that the kernel recovered at this doping level is much closer to four-fold symmetry than at lower cobalt dopings. This is consistent with previous measurements of the phase diagram of NaFe_1−*x*_Co_*x*_As^36^, including those by STM^37^ that have shown that the transition from orthorhombic to tetragonal symmetry happens before optimal doping. We see from the series of data in Fig. 5 that SBD-STM works over the entire doping range that is relevant to STM experiments and is able to recover high-quality kernels with phase-sensitive FTs.

From the results shown on both synthetic and real experimental STM data, we can see that SBD-STM provides a complete recovery of kernels in real space and therefore a phase sensitive recovery of the FT in reciprocal space. Within the formulation of QPI, the phase of the FT in the QPI signal is dependent on the incoming and outgoing quasiparticle’s Green’s function as well as the potential of the impurity. The availability of phase information can give us new insight into individual materials that is not available simply from the magnitude of the QPI signal. In the remainder of this paper, we consider one such new insight into the physics of NaFe_1−*x*_Co_*x*_As. NaFe_1−*x*_Co_*x*_As, like many of the pnictides displays a superconducting dome as a function of cobalt doping. The maximum *T*_c_ at optimal doping (*x* = 0.02) reaches 18 K. As with the other pnictides, determining the symmetry of the superconducting order parameter in this compound is of much current interest. In this context, recent theoretical work on QPI in the superconducting state of the pnictides^29,38,39^ has opened up the possibility of distinguishing different superconducting order parameters from their QPI signature. We follow the procedure outlined in the recent work by Altenfeld et al. ^39^, where the (real) Fourier transform of the QPI signal around a single defect *δρ*(**q**, *ω*) is integrated over all *q*-space to produce the quantity *δρ*(*ω*). This quantity is then anti-symmetrized with respect to energy relative to the Fermi level to produce a quantity *δρ*^−^(*ω*) = *δρ*(*ω*) − *δρ*(−*ω*). It is shown^29,39^ that in the case of *s*^±^ pairing, *δρ*^−^(*ω*) is large and of constant sign over the energy range near the superconducting gap. Conversely, in the case of *s*^++^ pairing, *δρ*^−^(*ω*) is expected to be small and have a sign change around the gap energy. In order to carry out the integration over **q** described in this procedure, it is required that we have access to the phase of the QPI signal. One way of achieving this is to directly image around an isolated dopant or defect where the complete phase sensitive pattern can be measured. Such STS imaging has recently been performed on iron chalcogenides^40,41^. However, this method has not been applied to the iron arsenides, especially at optimal doping where the defect or dopant density is high and the phase information in the FT was not previously available. Armed with the phase-sensitivity of SBD-STM, we now analyze differential conductance maps of near optimally doped NaFe_1−*x*_Co_*x*_As to investigate the superconducting order parameter at optimal doping.

**Fig. 6:**
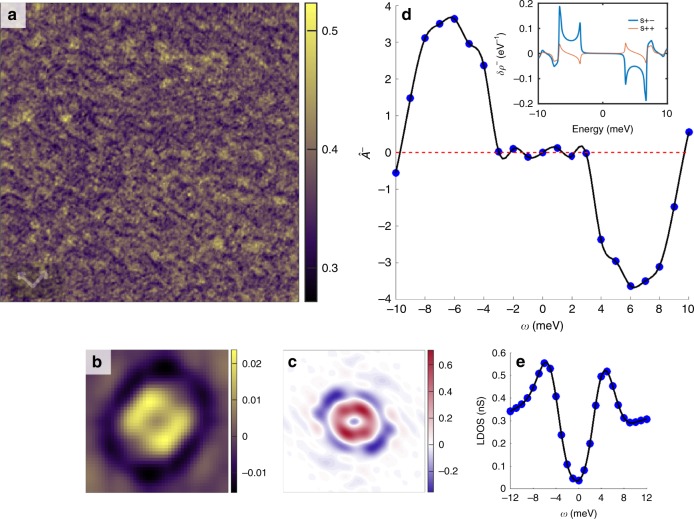
Utility of phase-sensitive QPI. **a** Differential conductance map at *ω* = −10 meV for an optimally doped NaFe_1−*x*_Co_*x*_As sample (*x* = 0.02), over a 250 nm × 250 nm^2^ field of view with *T* = 5.9 K, *I* = 200 pA, and *V*_*set*_ = −50 meV. **b** Recovered kernel, $\hat{{\mathcal{A}}}$, from the conductance map in **a**. **c** Fourier transform of the recovered kernel. **d** ${\hat{{\mathcal{A}}}}^{-}(\omega)$, computed from the recovered kernels. The large and constant sign of the response is an indication of *s*^±^ pairing. (inset) theoretical expectations for *s*^±^ and *s*^++^ pairing **e** Spatially averaged differential conductance from the same area as a function of energy, revealing the superconducting gap 2Δ = 11 meV. The STM spectroscopy maps have been drift corrected as outlined in Supplementary Note 7.

We start with a dataset that consists of 21 raw STS images from −10 to +10 meV in 1 meV increments on an optimally doped NaFe_1−*x*_Co_*x*_As sample at *T* = 5.9 K. One of these raw images at *ω* = −10 meV is shown in Fig. 6a (additional images are described in Supplementary Note 10). Notice that no individual motifs can be resolved by eye. We then proceed to recover the kernels $\hat{{\mathcal{A}}}({\bf{r}},\omega)$ at each energy using SBD-STM. An example of this recovery is shown in Fig. 6b, which is the recovered SBD-STM kernel $\hat{{\mathcal{A}}}$(*r*, *ω* = − 10 meV), recovered from the raw STS image in Fig. 6a. The recovered kernel lacks the strong anisotropy of recovered kernels from the underdoped regime, suggesting that electronic nematicity is not strong at this doping. Assuming that SBD-STM has worked correctly, the recovered $\hat{{\mathcal{A}}}({\bf{r}},\omega)$ is identical to the real-space QPI signal *δρ*(**r**, *ω*). We then take the real part of the 2-D Fourier transform $\hat{{\mathcal{A}}}({\bf{q}},\omega)$, as shown in Fig. 6c at *ω* = −10 meV. This FT has the full-phase information present, and we can then integrate over **q** and antisymmetrize with respect to energy:

5 $${\hat{{\mathcal{A}}}}^{-}(\omega)=\sum _{{\bf{q}}}{\mathrm{Re}}(\hat{{\mathcal{A}}}({\bf{q}},\omega))-{\mathrm{Re}}(\hat{{\mathcal{A}}}({\bf{q}},-\omega)).$$

We perform this procedure at each energy, and plot the resultant ${\hat{{\mathcal{A}}}}^{-}(\omega)$ in Fig. 6d. In Fig. 6e, we show the spatially averaged differential conductance from the same datasets as a function of energy, revealing the superconducting gap. From the two coherence peaks, we calculate a 2Δ of 11 meV. We can clearly see from Fig. 6d that ${\hat{{\mathcal{A}}}}^{-}(\omega)$ is peaked near the superconducting gap, and has no sign change in the energy range near the gap. For comparison we performed theoretical calculations of the anti-symmetrized correction to the LDoS, *δρ*^−^(*ω*) as shown in the inset of Fig. 6d following the original prescription^29^. Here, we use the electronic structure of Co-doped NaFeAs previously measured by angle-resolved photoemission spectroscopy (ARPES)^42,43^ and fitted to the 10 orbital tight-binding model^44^. The values of the superconducting gap on each band were taken to be Δ_h_ = 6.5 meV, Δ_e_ = 6.8 meV on the electron and the smaller hole pockets, respectively^42^, and Δ_H_ = 3.5 meV on the larger hole pocket^43^. Further details are given in Supplementary Note 9. As expected, the behavior of the ${\hat{{\mathcal{A}}}}^{-}(\omega)$ for the sign-changing gaps between electron and hole pockets follows the predicted behavior of the *δρ*^−^(*ω*) for an *s*^+−^-pairing state in which this quantity does not change in an energy range between the gaps on the hole and electron pockets, while for sign-preserving gaps this quantity is generally small, with an alternating sign between the gaps. A similar behavior is found in the experiment, as shown in the main Fig. 6d. This procedure illustrates some of the new physical insight into STS image data that can be obtained once the complete phase information in the QPI signal is available for analysis.

## Discussion

In its current implementation, SBD-STM addresses the problem of identifying a single motif across a series of images. Beyond the identification of real-space motifs in microscopy images, SBD-STM can also be applied to problems in which the motif is sparse in the appropriately chosen space, such as sparsity in the spatial gradient for natural image deblurring^45,46^. Moreover, the flexibility of the convolutional data model in (3) affords the natural generalization, ${\mathcal{Y}}={\sum }_{j=1}^{M}{{\mathcal{A}}}_{0}^{\left(j\right)} \otimes {{\mathcal{X}}}_{0}^{\left(j\right)}+{\mathcal{Z}}$, which expands the scope of SBD-STM to identify multiple distinct kernels in any series of images. In particular, STM images that contain various short-range orders, such as charge or spin density waves, would be amenable to a similar analysis^47–50^. SBD-STM recovered results from these STM measurements can be directly compared with theoretical predictions^51–53^ to understand the nature of competing orders in superconductors and other strongly correlated materials. Other analysis methodologies^47,50,54^ have been recently proposed to improve FT data fidelity and provide some phase-sensitive information on the structure of ordered phases. These alternative approaches provide compelling information under suitable conditions, but their results are still vulnerable to phase noise contamination. The correct implementation of SBD-STM to such cases remains an open but solvable problem.

## Supplementary information

Supplementary Information

## Data Availability

The datasets generated during and/or analysed during the current study are available from the corresponding author on reasonable request.
