# Supplementary material for: Dictionary learning in Fourier-transform scanning tunneling spectroscopy
Source: Nat Commun. 2020 Feb 26;11:1081. doi: 10.1038/s41467-020-14633-1 (PMC7044214; doi:10.1038/s41467-020-14633-1)
Supplement: Supplementary file 1 — Supplementary Information [file 41467_2020_14633_MOESM1_ESM.pdf]

Dictionary Learning in Fourier-Transform Scanning Tunneling Spectroscopy - Supplementary Information

Sky C. Cheung [a],[1] John Y. Shin [a],[1] Yenson Lau,[2] Zhengyu Chen,[2] Ju Sun,[2] Yuqian Zhang,[2] Marvin A. Müller,[3] Ilya M. Eremin,[3,4] John N. Wright,[2,†] and Abhay N. Pasupathy[1,‡]

[1]*Department of Physics, Columbia University, New York, NY 10027 USA*

[2]*Department of Electrical Engineering, Columbia University, New York, NY 10027 USA*

[3]*Institut für Theoretische Physik III, Ruhr-Universität Bochum, D-44801 Bochum, Germany*

[4]*National University of Science and Technology MISiS, 119049 Moscow, Russian Federation*

[a] These authors contributed equally to this work

[†] johnwright@ee.columbia.edu

[‡] pasupathy@phys.columbia.edu

Supplementary Table I. Summary of symbols and notation

Symbol	Description
$\mathbf{x}$	position: $\mathbb{R}^2$
$\mathbf{k}$	wavevector (Fourier dual to $\mathbf{x}$): $\mathbb{R}^2$
$\mathbf{q}$	relative wavevector, $\mathbf{k_f} - \mathbf{k_i}$: $\mathbb{R}^2$
ω	energy level (applied bias voltage): $\mathbb{R}$
$\rho(\mathbf{x}, \omega)$	LDoS/QPI map: $\mathbb{R}^2 \times \mathbb{R} \to \mathbb{R}$
$\rho_0(\mathbf{x}, \omega)$	LDoS/QPI map of an isolated defect: $\mathbb{R}^2 \times \mathbb{R} \to \mathbb{R}$
$\delta\rho(\mathbf{x}, \omega), \delta\rho_{\mathbf{0}}(\mathbf{x}, \omega)$	local variation of LDoS: $\mathbb{R}^2 \times \mathbb{R} \to \mathbb{R}$
$n \equiv n_1 \times n_2$	number of pixels in an $n_1 \times n_2$ measurement/image: $\mathbb{N}$
$m \equiv m_1 \times m_2$	number of pixels in an $m_1 \times m_2$ kernel: $\mathbb{N}$
s	number of observed energy levels (bias voltages): $\mathbb{N}$
$\mathcal{Y}$	observation of LDoS signature on a finite grid: $\mathbb{R}^{n_1 \times n_2 \times s}$
$\mathcal{A}$	kernel (from a single defect) on the grid: $\mathbb{R}^{m_1 \times m_2 \times s}$
$\mathcal{X}$	activation map on the grid: $\mathbb{R}^{n_1 \times n_2}$
$\mathcal{Z}$	additive noise
$\circledast$	convolution operator between each kernel slice and the activation map
Hat: $\hat{\mathcal{A}}, \hat{\mathcal{X}}, \ldots$	estimates of the corresponding variables
$\lVert \cdot \rVert_F^2$	Frobenius norm: sum of the squared entries of the given variable
$\mathcal{S}$	the sphere $\{\mathcal{A} : \lVert \mathcal{A} \rVert_F = 1\}$
$\psi_\lambda(\mathcal{A}, \mathcal{X})$	objective for fixed λ and given $(\mathcal{A}, \mathcal{X})$
$\varphi_\lambda(\mathcal{A})$	marginalized objective for fixed λ and given $\mathcal{A}$: $\varphi_\lambda(\mathcal{A}) = \min_{\mathcal{X}} \psi_\lambda(\mathcal{A}, \mathcal{X})$
η	additive Gaussian noise variance
$\mathrm{SNR} \equiv \frac{var(\mathcal{A})}{\eta}$	signal-to-noise ratio
$\epsilon(\mathcal{A}, \hat{\mathcal{A}})$	error metric: angle between $\mathcal{A}$ and $\hat{\mathcal{A}}$ on the hemisphere
θ	kernel concentration: probability that a pixel of $\mathcal{X}$ is a defect center

SUPPLEMENTARY NOTE 1: Simulated STM Spectroscopic Measurements on a Square Lattice

We consider a two-dimensional structure of atoms forming an infinite periodic square array, with lattice constant a. Under the single-electron Tight-Binding (TB) approximation, electrons are localized to fixed atomic sites [1–3]. This model allows for an inter-site interaction consisting of a hopping integral between nearest-neighboring lattice sites, characterized as the fixed hopping-parameter $t < 0$. The on-site energies of the lattice are assumed to be a constant E_0. Introducing point defects into the system breaks translational symmetry of the system and perturbs the Local Density of States (LDoS). These point defects are assumed to have an energy of E_j, where j enumerates the number N_d of defects.

We seek to calculate the LDoS $\rho(\mathbf{x}, \omega)$ for this system with a finite number of point defects.

$\rho(\mathbf{x},\omega)$ represents the number of states that are available to be occupied by electrons – a measure of the relative probability of finding an electron at location $\mathbf{x} \in \mathbb{R}^\mathbf{2}$ with energy $\omega \in \mathbb{R}$. In STM spectroscopy [4], $\rho(\mathbf{x},\omega)$ is recorded as a function of the probe tip location $\mathbf{x}$ and an applied bias voltage ω between the tip and specimen.

Perturbation Theory

The LDoS is determined by the matrix elements of the Green's Function (GF) in the coordinate representation:

$$\rho(\mathbf{x},\omega) = \frac{\mathbf{-1}}{\pi}\,\mathrm{Im}\left[\langle \mathbf{x}|\,\hat{\mathbf{G}}\,|\mathbf{x}\rangle\right] \tag{1}$$

with the GF defined as $\hat{G}(\omega) = (\omega - \hat{H})^{-1}$.

Assuming no direct coupling between the lattice and point defects, the system Hamiltonian $\hat{H}$ can be expressed as the sum of two contributions $\hat{H} = \hat{H}_0 + \hat{H}_1$ where $\hat{H}_0$ is the TB Hamiltonian for a square lattice and $\hat{H}_1$ is the impurity Hamiltonian:

$$\hat{H}_1 = \sum_{\alpha=1}^{N_d} E_\alpha \,|\alpha\rangle\,\langle\alpha|$$

in which α enumerates the N_d point defects, located at distinct positions $\mathbf{x}_\alpha \in \mathbb{R}^\mathbf{2}$.

Since $\hat{H}_0$ can be easily diagonalized in $\mathbf{k}$-momentum space, we treat $\hat{H}_1$ as a perturbation on $\hat{H}_0$. In accordance with perturbative scattering theory, the GF can be expressed as:

$$\hat{G} = \hat{G}_0 + \hat{G}_0\hat{T}\hat{G}_0 \tag{2}$$

where $\hat{G}_0$ and $\hat{T}$ are the Bare Lattice Green's Function (BLGF) and the scattering T-matrix, respectively, which satisfy the following:

$$\hat{G}_0(\omega) = \frac{1}{\omega - \hat{H}_0} \tag{3}$$

$$\hat{T} = \hat{H}_1\left(\hat{I} - \hat{G}_0\hat{H}_1\right)^{-1} \tag{4}$$

Reduction to Matrix Elements

Following the prescription in Supplementary Equation (1), we compute the matrix elements of $\hat{G}$ from Supplementary Equation (2) to obtain:

$$\rho(\mathbf{x},\omega) = \rho^{(\mathbf{0})}(\mathbf{x},\omega) + \frac{\mathbf{-1}}{\pi}\,\mathrm{Im}\left[\langle \mathbf{x}|\,\hat{\mathbf{G}}_\mathbf{0}\hat{\mathbf{T}}\hat{\mathbf{G}}_\mathbf{0}\,|\mathbf{x}\rangle\right]$$

where $\rho^{(0)}(\mathbf{x},\omega)$ is the LDoS for a system with no defects. Since experimental probes, such as STM, are sensitive to changes in the LDoS, rather than the LDoS itself, we will treat

$\delta\rho(\mathbf{x},\omega) \equiv \rho(\mathbf{x},\omega) - \rho^{(\mathbf{0})}(\mathbf{x},\omega)$ as the physical observable:

$$\delta\rho(\mathbf{x},\omega) = \frac{\mathbf{-1}}{\pi}\,\mathrm{Im}\left[\langle\mathbf{x}|\,\hat{\mathbf{G}}_{\mathbf{0}}\hat{\mathbf{T}}\hat{\mathbf{G}}_{\mathbf{0}}\,|\mathbf{x}\rangle\right] = \frac{\mathbf{-1}}{\pi}\,\mathrm{Im}\left[\sum_{\alpha,\beta=\mathbf{1}}^{\mathbf{N_d}}\mathbf{G_0}(\mathbf{x},\mathbf{x}_\alpha)\,\mathbf{T}_{\alpha,\beta}\,\mathbf{G_0}(\mathbf{x}_\beta,\mathbf{x})\right] \tag{5}$$

with the following defined quantities:

$$G_0(\mathbf{x},\mathbf{y}) = \langle\mathbf{x}|\,\hat{G}_0\,|\mathbf{y}\rangle \tag{6}$$

$$T_{\alpha,\beta} = \left\langle\alpha\middle|\hat{T}\middle|\beta\right\rangle \tag{7}$$

Henceforth, we will refer to $\delta\rho(\mathbf{x},\omega)$ inSupplementary Equation (5) as the LDoS and vice-versa.

Calculation of Matrix Elements

The following section discusses the calculation of matrix elements of $\hat{G}_0$ and $\hat{T}$ in Supplementary Equation (6) and Supplementary Equation (7) necessary to determine $\delta\rho(\mathbf{x},\omega)$ in Supplementary Equation (5).

<u>Bare Lattice Green's Function Matrix Elements</u>

We aim to compute the matrix elements of the BLGF in the coordinate representation $G_0(\mathbf{x},\mathbf{x}';\omega)$. Starting with the definition of the BLGF inSupplementary Equation (3), we have:

$$\hat{G}_0 = \int_{\mathrm{BZ}} \mathrm{d}\mathbf{k}\frac{\mathbf{1}}{\omega - \hat{\mathbf{H}}_{\mathbf{0}}}\,|\mathbf{k}\rangle\langle\mathbf{k}| = \int_{\mathrm{BZ}} \mathrm{d}\mathbf{k}\frac{\mathbf{1}}{\omega - \mathbf{E_k}}\,|\mathbf{k}\rangle\langle\mathbf{k}| \tag{8}$$

where the integration is across the first Brillouin Zone (BZ). The energy dispersion $E_{\mathbf{k}}$ of the square lattice is:

$$E_{\mathbf{k}} = E_0 - 2t\left(\cos(k_1 a) + \cos(k_2 a)\right) \tag{9}$$

SubstitutingSupplementary Equation (9) into Supplementary Equation (8) gives the BLGF coordinate representation matrix elements:

$$\begin{aligned}\langle\mathbf{x}|\,\hat{G}_0\,|\mathbf{x}'\rangle &= \frac{1}{(2\pi)^2}\int_{\mathrm{BZ}}\mathrm{d}\mathbf{k}\,\mathbf{e}^{\mathbf{ik\cdot(x-x')}}\frac{\mathbf{1}}{\omega - \mathbf{E_0} - \mathbf{2t}\left(\cos(\mathbf{k_1 a}) + \cos(\mathbf{k_2 a})\right)} \\ G_0(\mathbf{x},\mathbf{x}') &= \frac{1}{(2\pi)^2}\frac{1}{2t}\int_{\mathrm{BZ}}\mathrm{d}\mathbf{k}\frac{\mathbf{e}^{\mathbf{ik_1(x_1-x_1')}}\mathbf{e}^{\mathbf{ik_2(x_2-x_2')}}}{\mathbf{b} - \left(\cos(\mathbf{k_1 a}) + \cos(\mathbf{k_2 a})\right)}\end{aligned} \tag{10}$$

where $b \equiv \frac{\omega - E_0}{2t}$ is a dimensionless parameter with a complex $\omega \to \omega + i\epsilon$ analytic continuation. Defining the normalized position deviations as $s_j \equiv \frac{1}{a}(x_j - x_j')$,Supplementary Equation (10) can be reduced to quadratures:

$$G_0(\mathbf{x},\mathbf{x}') = \frac{1}{(2\pi)^2}\frac{1}{2t}\int_{-\frac{\pi}{a}}^{\frac{\pi}{a}}\int_{-\frac{\pi}{a}}^{\frac{\pi}{a}}\frac{\mathrm{d}k_1\,\mathrm{d}k_2\,e^{ik_1as_1}e^{ik_2as_2}}{b-(\cos(k_1a)+\cos(k_2a))}$$
$$G_0(\mathbf{x},\mathbf{x}') = \frac{1}{(2\pi)^2}\frac{1}{2t}\frac{4}{a^2}\int_0^{\pi}\int_0^{\pi}\mathrm{d}\phi_1\,\mathrm{d}\phi_2\frac{\cos(\phi_1 s_1)\cos(\phi_2 s_2)}{b-\cos\phi_1-\cos\phi_2}$$
$$G_0(\mathbf{x},\mathbf{x}') = \frac{1}{(2\pi)^2}\frac{1}{2t}\frac{4}{a^2}I_{\mathrm{sq}}\left(\frac{x_1-x_1'}{a},\frac{x_2-x_2'}{a},b\right) \tag{11}$$

where we define the 2-dimensional definite integral I_{sq} as:

$$I_{\mathrm{sq}}(s_1,s_2,b) \equiv \int_0^{\pi}\int_0^{\pi}\mathrm{d}\phi_1\,\mathrm{d}\phi_2\frac{\cos(s_1\phi_1)\cos(s_2\phi_2)}{b-\cos\phi_1-\cos\phi_2} \tag{12}$$

Scattering T-Matrix Elements

We are also interested in computing the matrix elements of $\hat{T}$ between defects, as described inSupplementary Equation (7). Recalling that E_α is the on-site energy of the defect located at $\mathbf{x}_\alpha$, the defect-defect matrix elements ofSupplementary Equation (4) are:

$$T_{\alpha,\beta} = \langle\alpha|\,\hat{H}_1\left(\hat{I}-\hat{G}_0\hat{H}_1\right)^{-1}|\beta\rangle$$
$$= E_\alpha\,\langle\alpha|\left(\hat{I}-\hat{G}_0\hat{H}_1\right)^{-1}|\beta\rangle$$
$$T_{\alpha,\beta} = E_\alpha\left(\delta_{\alpha,\beta}-E_\beta\,G_0(\mathbf{x}_\alpha,\mathbf{x}_\beta)\right)^{-1} \tag{13}$$

The scattering T-matrix elements are completely determined bySupplementary Equation (13) once the BLGF matrix elements are obtained inSupplementary Equation (11).

Numerical Results on a Single Point Impurity Embedded within the $T_{\alpha,\beta}$ calculation inSupplementary Equation (13) is a matrix inverse involving the matrix elements of $\hat{G}_0$ corresponding to inter-defect scatterings. However if we consider the $N_d = 1$ single impurity limit, we can bypass the formal matrix inversion. In this limit, the scattering T-matrix becomes a scalar

$$T(\omega) = \frac{1}{E_1^{-1}-G_0(\mathbf{x_d},\mathbf{x_d};\omega)} \tag{14}$$

where the impurity location is $\mathbf{x_d} \in \mathbb{R}^{\mathbf{2}}$. SubstitutingSupplementary Equation (14) into Supplementary Equation (5) gives $\delta\rho(\mathbf{x},\omega)$ in the presence of a single point impurity:

$$\delta\rho(\mathbf{x},\omega) = \frac{-1}{\pi}\,\mathrm{Im}\left[\frac{G_0(\mathbf{x},\mathbf{x_d};\omega)\mathbf{G_0}(\mathbf{x_d},\mathbf{x};\omega)}{E_1^{-1}-G_0(\mathbf{x_d},\mathbf{x_d};\omega)}\right]$$
$$\delta\rho(\mathbf{x},\omega) = \frac{-1}{\pi}\,\mathrm{Im}\left[\frac{G_0^2(\mathbf{x},\mathbf{x_d};\omega)}{E_1^{-1}-G_0(\mathbf{x_d},\mathbf{x_d};\omega)}\right] \tag{15}$$

To produce simulated single-defect STM measurements to assess the SBD-STM approach, $\delta\rho(\mathbf{x},\omega)$ was computed at all measurement positions $\mathbf{x}$ for a particular fixed value of defect energy E_d, hopping parameter t, measurement energy ω, and specified defect location $\mathbf{x_d} = \mathbf{0}$. Conforming with typical STM experimental datasets, the measurement grid consisted of 256×256 equally-spaced points chosen to overlap with the square lattice containing N^2 atomic sites, with $N = 50$. $\delta\rho(\mathbf{x},\omega)$ was numerically computed at every grid point at 41 different energy ω values. Typical LDoS maps of single-defect square lattice systems are shown in Supplementary Figure 1.

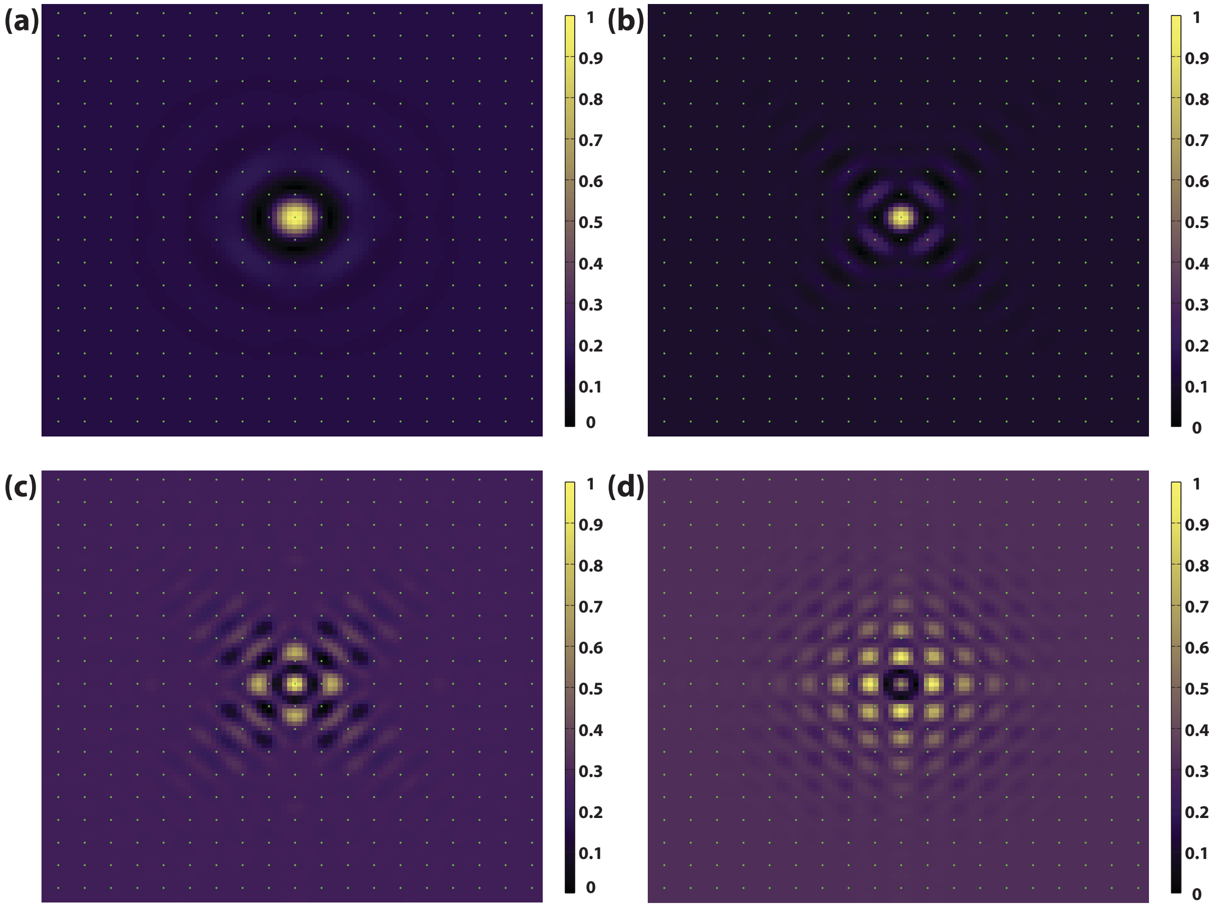

Supplementary Figure 1. $\delta\rho(\mathbf{x},\omega)$ for a single impurity on a square lattice with $t = -0.2$ and $\omega = -0.5$ (a), 0 (b), +0.2 (c), and +0.35 (d). All LDoS maps are normalized between 0 and 1. The arrays of green dots are guides to the eyes indicating the atomic positions of the square lattice.

One observes that the LDoS resulting from a single impurity has a structure entangled with the underlying atomic lattice. As the probe energy ω changes, the LDoS signatures also modulate in the vicinity of the defect. Far away from the defect ($\gtrsim 10$ atomic lengths), $\delta\rho(\mathbf{x},\omega)$ is nearly constant.

Numerical Results on Multiple Point Defects Simulations on square lattice systems possessing multiple point defects have also been implemented usingSupplementary Equation (5). Shown in Supplementary Figure 2 are representative calculated LDoS maps at different energies for 70 point impurities randomly distributed on the square lattice. As expected, the effect of multiple defect scattering is more vivid at locations where the defects are clustered together.

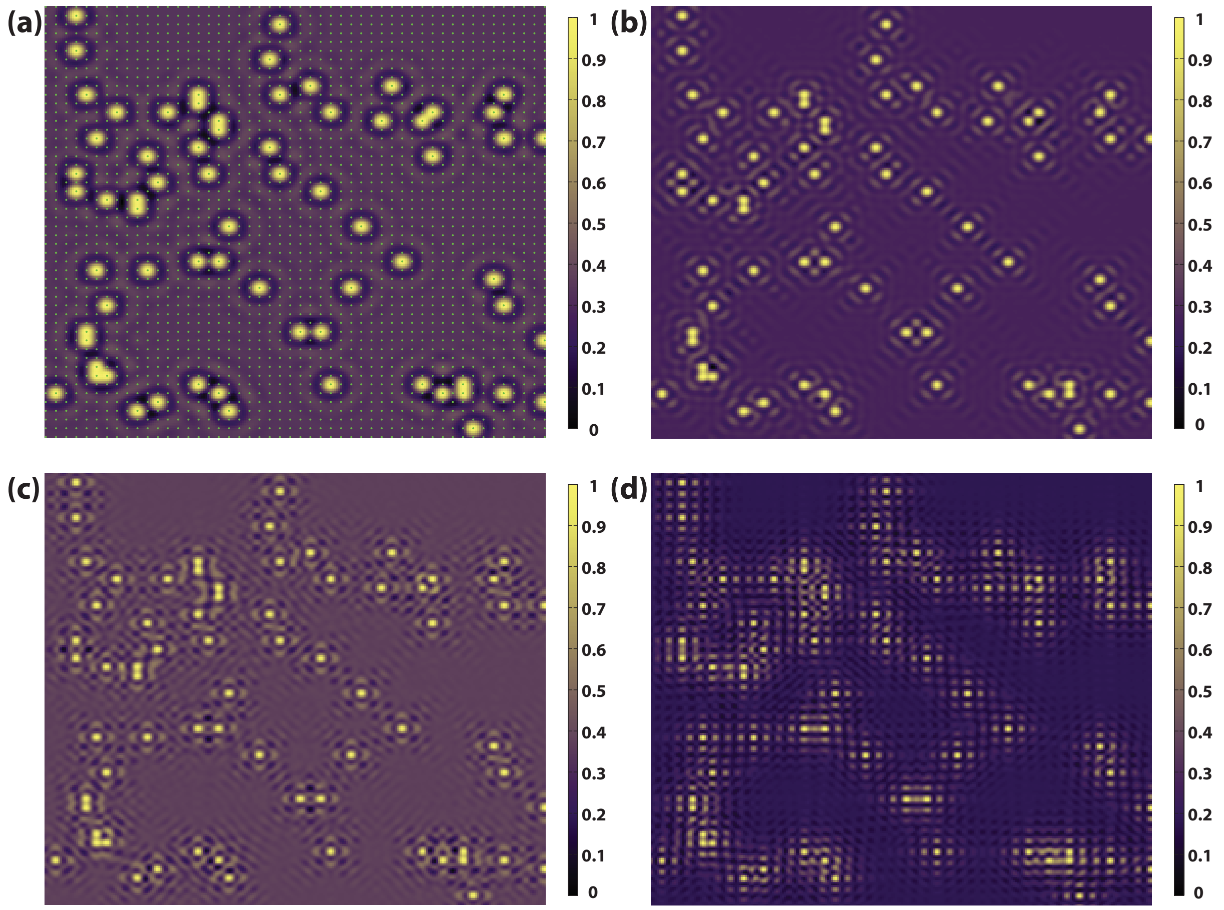

Supplementary Figure 2. $\delta\rho(\mathbf{x}, \omega)$ for 70 impurities randomly distributed on a 50×50 atom square lattice with $t = -0.2$ and $\omega = -0.5$ (a), 0 (b), +0.2 (c), and +0.35 (d). All LDoS maps are normalized between 0 and 1. The array of green dots in (a) is a guide to the eyes indicating the atomic positions of the square lattice. Point defects are located at the same atomic positions.

SUPPLEMENTARY NOTE 2: Sparse Inverse Problems

The convolutional model proposed in equation (1) of the main text provides a concise description of STM datasets. However, the problem of inferring $\mathcal{A}_0$ and $\mathcal{X}_0$ from $\mathcal{Y}$ alone is ill-posed in general – for $s \in \mathbb{N}$ voltage slices, one must extract $n_1 n_2 + s \cdot m_1 m_2$ values from $s \cdot n_1 n_2$ observed pixels. Even with many voltage slices, our kernel slices from $\mathcal{A}_0$ are

approximately low-pass and vary slowly across bias voltages, so the problem of recovering $\mathcal{X}_0$ would remain ill-posed even if $\mathcal{A}_0$ were known a priori. Since our problem involves inferring both $\mathcal{A}_0$ and $\mathcal{X}_0$, it is essential to incorporate well-motivated assumptions about the structure of the data.

In this work, we capitalize on the *sparsity* of $\mathcal{X}_0$ to render the problem well-posed. Informally, $\mathcal{X}_0$ is sparse if most of its entries are zero. The sparse signal model is applicable whenever one is interested in signals that are composed of a few components from a large "dictionary": although one can find defects at any location in $\mathcal{X}_0$ within the convolutional model, the expected number of defects in typical STM datasets is much smaller than the number of pixels in the image.

The power of sparsity is most clearly illustrated within the context of *linear inverse problems*. For instance, suppose one is given a matrix $\mathbf{D} \in \mathbb{R}^{m\times n}$ and an observation $\mathbf{y} = \mathbf{D}\mathbf{x}_0$, and wishes to recover $\mathbf{x}_0$. When $m \ll n$, this is an ill-posed problem with infinitely many solutions. However, if $\mathbf{x}_0$ is known to be sparse, we can attempt to recover $\mathbf{x}_0$ by solving the following optimization problem, which seeks the *sparsest* solution to the underdetermined linear system $\mathbf{y} = \mathbf{D}\mathbf{x}$:

$$\begin{cases} \underset{\mathbf{x}}{\text{minimize}} & \|\mathbf{x}\|_0 \\ \text{subject to} & \mathbf{D}\mathbf{x} = \mathbf{y}. \end{cases} \tag{16}$$

Here the ℓ_0-"norm" $\|\mathbf{x}\|_0$ counts the number of nonzero entries in the vector $\mathbf{x}$. Under very general conditions, the unique optimal solution toSupplementary Equation (16) is $\mathbf{x}_0$, the signal that generated the observation $\mathbf{y}$. For example, for a generic choice of $\mathbf{D}$, $\mathbf{x}_0$ is the unique optimal solution toSupplementary Equation (16), provided it has nonzero entries, k, that are less than half the number of observations $m > 2k$. In this situation, imposing sparsity constraints renders an ostensibly ill-posed inverse problem well-posed [5].

The optimization inSupplementary Equation (16) is purely conceptual in nature: it involves a search over all possible sets of nonzero entries for the vector $\mathbf{x}$, and hence is intractable even for small m and n. To address this issue, many computationally efficient heuristics have been developed as alternatives to ℓ_0-norm minimization for computing sparse solutions. One popular heuristic is to *relax*Supplementary Equation (16) by replacing the ℓ_0-norm $\|\mathbf{x}\|_0$ with the ℓ_1-norm $\|\mathbf{x}\|_1 = \sum_{i=1}^{n} |x_i|$, leading to a convex optimization problem

known as *basis pursuit*:

$$\begin{cases} \underset{\mathbf{x}}{\text{minimize}} & \|\mathbf{x}\|_1 \\ \text{subject to} & \mathbf{D}\mathbf{x} = \mathbf{y}. \end{cases} \tag{17}$$

This problem can be converted to a linear program, and hence solved in polynomial time using general-purpose tools. A variety of dedicated algorithms are also available forSupplementary Equation (17), which can efficiently solve very large instances to an accuracy acceptable for modern statistical and signal processing applications. In short, replacing the ℓ_0-norm with the ℓ_1-norm yields a problem that we can efficiently solve, at scale, on contemporary hardware.

However, a crucial question arises: *have we lost anything in moving from the problem that we would like to solve (ℓ_0 minimization) to a problem that we can solve (ℓ_1 minimization)?* Specifically, does ℓ_1 minimization still recover the sparse signal $\mathbf{x}_0$? The answer to the second question is a qualified "yes." Perhaps surprisingly, tractable ℓ_1 minimizations also exactly recover $\mathbf{x}_0$, provided that (i) $\mathbf{D}$ is sufficiently "nice," and (ii) $\mathbf{x}_0$ is sufficiently sparse. For example, for generic (random) $\mathbf{D}$, if $\mathbf{x}_0$ has at most k nonzero entries, basis pursuit succeeds when

$$m \geq C \cdot k \cdot \log(n/k), \tag{18}$$

in which C is a fixed constant [6, 7]. If we consider m as the number of measurements we have observed, this recovery rate is nearly optimal: $\mathbf{x}_0$ has k nonzero entries, and it can be efficiently recovered from about $k \log n$ measurements. These results hold for random $\mathbf{D}$, but deterministic results are also known – the required property, coarsely stated, is that sparse vectors $\mathbf{x}$ cannot be too close to the nullspace of $\mathbf{D}$. This requirement makes intuitive sense: any sparse vector $\mathbf{x}$ that produces a measurement very close to zero ($\mathbf{y} = \mathbf{D}\mathbf{x} \simeq \mathbf{0}$) will become very difficult to recover, especially in the presence of noise.

If measurements are contaminated with zero-mean additive noise, i.e. $\mathbf{y} = \mathbf{D}\mathbf{x}_0 + \mathbf{w}$, then a popular choice for estimating $\mathbf{x}_0$ involves solving the LASSO problem [8],

$$\min_{\mathbf{x}} \underbrace{\tfrac{1}{2}\|\mathbf{D}\mathbf{x} - \mathbf{y}\|_2^2}_{\text{data fidelity}} + \lambda \underbrace{\|\mathbf{x}\|_1}_{\text{sparsity}}. \tag{19}$$

This formulation balances between a standard least squares term, which ensures fidelity to the observed data, and an ℓ_1-norm *regularizer*, which biases $\mathbf{x}$ towards sparse solutions.

When $\mathbf{D}$ is randomly chosen, Ref. 9 asserts that – with similar sampling requirements toSupplementary Equation (18) – solvingSupplementary Equation (19) will recover the correct sign pattern from $\mathbf{x}_0$, provided that λ and the entries of $\mathbf{x}_0$ are large enough to overcome the bias introduced by the regularizer $\|\mathbf{x}\|_1$ and the variance of the noise term.

Theoretical guarantees of this nature inform the algorithmic design for applications in science and engineering under a variety of settings; we refer the interested reader to Refs. 10 and 11 and the text Ref. 12. For example, in biomedical imaging, these efforts help establish performance and resource requirements in systems where minimal imaging time or radiation dosage are of critical importance [13].

The Sparse Convolutional Model for the STM datasets

In this section, we show how to express the STM model, presented in equation (1) of the main text, as a convolution between a defect signature and a sparse activation map, and describe how to estimate the sparse activation map using the LASSOSupplementary Equation (19). In this formulation, $\mathbf{D}$ plays the role of the convolution with the defect signature, and $\mathbf{x}$ represents the sparse activation map. Previously, $\mathbf{D}$ was assumed to be known beforehand, but in STM data analysis, we must simultaneously estimate $\mathbf{D}$ and $\mathbf{x}$. In the next section, we will describe the technical challenges associated with this harder problem, and offer an efficient algorithm that accurately estimates both $\mathbf{D}$ and $\mathbf{x}$, on well-structured numerical examples.

Restricting the STM image and the defect locations $\mathbf{x_j}$ to an infinitely large grid of pixel locations $\mathbb{Z}^2$ and using γ to denote the Kronecker delta so that $\gamma(\mathbf{u}) = \mathbf{1}$ if $\mathbf{u} = \mathbf{0}$ and $\gamma(\mathbf{u}) = 0$ elsewhere, we have:

$$\delta\rho(\mathbf{x}, \omega) = \sum_{\mathbf{j=1}}^{\mathbf{N}} \mathbf{c_j} \cdot \delta\rho_{\mathbf{0}}(\mathbf{x} - \mathbf{x_j}, \omega) = \sum_{\mathbf{u} \in \mathbb{Z}^{\mathbf{2}}} \delta\rho_{\mathbf{0}}(\mathbf{x} - \mathbf{u}, \omega) \cdot \underbrace{\left(\sum_{j=1}^{N} c_j \cdot \gamma(\mathbf{u} - \mathbf{x_j}) \right)}_{\text{collect spikes}}.$$

Collecting the defect locations into $\Gamma(\mathbf{x}) \equiv \sum_{\mathbf{j=1}}^{\mathbf{N}} \mathbf{c_j} \gamma(\mathbf{x} - \mathbf{x_j})$ leads to the *convolution sum* [14] between $\delta\rho_0$ and Γ,

$$\delta\rho(\mathbf{x}, \omega) = \sum_{\mathbf{u} \in \mathbb{Z}^{\mathbf{2}}} \delta\rho_{\mathbf{0}}(\mathbf{x} - \mathbf{u}, \omega) \cdot \mathbf{\Gamma}(\mathbf{u}) = \big(\delta\rho_{\mathbf{0}} * \mathbf{\Gamma}\big)(\mathbf{x}, \omega). \tag{20}$$

Naturally, mild assumptions are needed on the sizes of the observation $\delta\rho$, defect locations $\mathbf{x_j}$, and LDoS signature $\delta\rho_0$. Letting $W_n = \{0, \ldots, n_1 - 1\} \times \{0, \ldots, n_2 - 1\}$ be the

observation window, we assume that $\delta\rho(\mathbf{x},\omega) = \mathbf{0}$ for any $\mathbf{x} \notin \mathbf{W_n}$, and $\bigcup_j \mathbf{x_j} \subseteq \mathbf{W_n}$, i.e. the observations and defect locations are bounded within an $n_1 \times n_2$ window. We also assume that $m_1 \ll n_1$, and $m_2 \ll n_2$ so that the individual defect signature takes up a relatively small portion of the observation window W_m.

Denoting the discretized versions of the activation map, the LDoS signature of a single impurity, and the full STM image – with energies discretized to s levels – by $\mathcal{X}_0 \in \mathbb{R}^{n_1\times n_2}$, $\mathcal{A}_0 \in \mathbb{R}^{n_1\times n_2\times s}$, and $\mathcal{Y} \in \mathbb{R}^{m_1\times m_2\times s}$ respectively, we have

$$\mathcal{Y}(\cdot,\omega) = \mathcal{A}_0(\cdot,\omega) * \mathcal{X}_0,$$

which we express concisely as $\mathcal{Y} = \mathcal{A}_0 \circledast \mathcal{X}_0$. $\mathcal{X}_0$ is expected to be sparse in this formulation, otherwise the observation would be saturated with defects. Since the convolution operator $\circledast$ is linear with respect to each argument, a LASSO problem can be solved to produce a sparse estimate of $\mathcal{X}_0$ from a noisy observation, provided that $\mathcal{A}_0$ is known:

$$\hat{\mathcal{X}} \leftarrow \min_{\mathcal{X}} \tfrac{1}{2}\|\mathcal{A}_0 \circledast \mathcal{X} - \mathcal{Y}\|_F^2 + \lambda\|\mathcal{X}\|_1 \tag{21}$$

This is an example of a *Sparse Deconvolution* (SD) problem. (Recall that the *Frobenius norm* $\|\cdot\|_F^2$ is the sum of square entries in the tensorial setting.)

On the contrary, $\mathcal{A}_0$ is unknown in the STM setting, leading to a bilinear inverse problem known as *Blind Deconvolution* (BD). However, assuming that a good approximation of the observation $\mathcal{Y}$ can only be produced by convolving a sparse activation map $\mathcal{X}$ with a candidate kernel $\mathcal{A}$ once $\mathcal{A} \simeq \mathcal{A}_0$, the optimal objective value from (21) may serve as a basis for finding $\mathcal{A}$ by formulating the problem

$$(\hat{\mathcal{A}}, \hat{\mathcal{X}}) \leftarrow \min_{\mathcal{A}} \min_{\mathcal{X}} \tfrac{1}{2}\|\mathcal{A} \circledast \mathcal{X} - \mathcal{Y}\|_F^2 + \lambda\|\mathcal{X}\|_1 \tag{22}$$

as an instance of the *Sparse Blind Deconvolution* (SBD) problem. This problem is nonconvex, and consequently, characterizing the performance of efficient algorithms is challenging: currently available theory does not completely explain the good behavior of simple nonconvex methods on practical problems. Nevertheless, this problem is part of a rapidly developing area of study with practical and theoretical implications.

Relevant problems and literature

Recently, variants of the deconvolution and BD problems have attracted significant theoretical interest. For instance, Refs. 15–17 study the solvability of BD problems under various

settings. However strong assumptions are often needed, and the settings studied thus far do not staunchly align with our SBD settingSupplementary Equation (22).

While the difficulty of establishing theory for nonconvex optimization makes guarantees for SBD problem difficult to formulate, many of the same concerns in existing literature motivate our SBD-STM algorithm and its analysis: the effect of various sparsity levels, noise power, and the choice of tradeoff parameter λ are motivated by studies involving the LASSO problemSupplementary Equation (19).

Studies involving variants of the SD problem are more extensive, and provide an important source of intuition for the SBD problem. For example, Ref. 18 suggests that even when $\mathcal{A}_0$ is known in advance, the recoverability of the *activation locations* depends strongly on the distance between these locations in $\mathcal{X}_0$ as well as the conditioning of $\mathcal{A}_0$ and the noise level. As a result, it is difficult to expect perfect recovery of the defect locations from $\hat{\mathcal{X}}$, especially when the kernels in $\mathcal{A}_0$ are approximately low-pass and under copious noise.

Practically, deconvolution problems are of interest in a large variety of fields. The extraction of spike signals in neuroscience [19] and the deblurring of images [20] serve as quintessential examples of problems that rely critically on a (blind) deconvolutional model. We refer interested readers to Refs. 21 and 22 for more details.

SUPPLEMENTARY NOTE 3: Solving the SBD-STM Problem

Symmetries and Nonconvexity

The bilinearity of the convolutional model $\mathcal{Y} = \mathcal{A}_0 \circledast \mathcal{X}_0$ leads to a number of difficulties characterizing or solving the problemSupplementary Equation (22). One class of difficulties arises due to symmetry, in the sense that there are many distinct choices of $(\mathcal{A}, \mathcal{X})$ that are equally sparse *and* approximate $\mathcal{Y}$ equally well. Having to work with entire equivalence classes of $(\mathcal{A}, \mathcal{X})$ introduces computational issues and makes characterizing our solution quality of the SBD-STM problem difficult, so modifications for breaking such symmetries are needed. The methods used to resolve undesirable phenomena arising from such symmetries lead to many sources of nonconvexity that require special consideration for yielding robust and reliable estimates of $\mathcal{A}_0$ and $\mathcal{X}_0$.

Optimization on the sphere: One consequence of bilinearity is *scaling symmetry*: for any $\mathcal{A}$, $\mathcal{X}$, and scalar $\alpha \in \mathbb{R}$ scaling one variable up and the other down by α leads to the same convolution, i.e. $\mathcal{A} \circledast \mathcal{X} = (\alpha\mathcal{A}) \circledast (\alpha^{-1}\mathcal{X})$. Because the ℓ_1-norm is a relaxation of the ℓ_0-norm and not a true sparsity measure, solutions that essentially have the same data fidelity and

sparsity can lead to wildly different objective values through rescaling by α. This undesirable trait is addressed by fixing $\mathcal{A}$ to lie on the sphere $\mathcal{S} = \{\mathcal{A} \in \mathbb{R}^{m_1 \times m_2 \times s} : \|\mathcal{A}\|_F = 1\}$, which greatly restricts the equivalence class due to scaling symmetry to that of sign flips $\mathcal{A} \circledast \mathcal{X} = (-\mathcal{A}) \circledast (-\mathcal{X})$. However, the optimization problem must now be solved over a nonconvex manifold $\mathcal{S}$, requiring modifications to standard optimization tools so that operations applied to produce updates are consistent with the geometry of $\mathcal{S}$.

Shifting symmetry produces local minima: The convolution sum contains a *shifting symmetry* when operating on functions over $\mathbb{Z}^2$: shifting the activation map and the LDoS signature by $\mathbf{\Delta} = (\mathbf{\Delta_1}, \mathbf{\Delta_2})$ pixels in opposite directions yields the same observation. From-Supplementary Equation (20), observe that

$$\begin{aligned}
\delta\rho(\mathbf{x}, \omega) &= (\delta\rho_0 * \Gamma)(\mathbf{x}, \omega) \\
&= \sum_{\mathbf{u} \in \mathbb{Z}^2} \delta\rho_0(\mathbf{x} - \mathbf{u}, \omega) \cdot \mathbf{\Gamma}(\mathbf{u}) \\
&= \sum_{\mathbf{u} \in \mathbb{Z}^2} \delta\rho_0\left((\mathbf{x} - \mathbf{u}) + \mathbf{\Delta}, \omega\right) \cdot \Gamma\left(\mathbf{u} - \mathbf{\Delta}\right) \\
\delta\rho(\mathbf{x}, \omega) &= \mathcal{T}\left[\delta\rho_0; \mathbf{\Delta}\right](\mathbf{x}, \omega) * \ \mathcal{T}\left[\mathbf{\Gamma}; -\mathbf{\Delta}\right](\mathbf{x}, \omega)
\end{aligned}$$

where the shift-translation operator $\mathcal{T}$ of a function $f(\mathbf{x})$ is defined as: $\mathcal{T}\left[f; \mathbf{\Delta}\right](\mathbf{x}) \equiv \mathbf{f}(\mathbf{x} + \mathbf{\Delta})$.

The restriction of $\delta\rho$ to the size of $\mathcal{A}$ breaks this symmetry, since the shift $(\mathcal{T}\left[\mathcal{A}_0; \mathbf{\Delta}\right], \mathcal{T}\left[\mathcal{X}_0; -\mathbf{\Delta}\right])$ from $(\mathcal{A}_0, \mathcal{X}_0)$ would yield worse data fidelity if any signal from $\delta\rho$ were shifted outside the size of $\mathcal{A}$. On the other hand, this restriction condition is very weak: the loss in data fidelity can be insignificant when the window sizes are large. Indeed, such *shift-truncations* are often achieved by methods employed to solve problems similar to Supplementary Equation (22) – we discuss attempts to refine solutions from shift-truncations further in Section .

As a result of the sign and shifting symmetries, objectives related toSupplementary Equation (22) possess several local minima in the form of *signed shift-truncations*, making the objective function nonconvex as demonstrated in Supplementary Figure 3. In general, nonconvex problems are notorious for local minima or undesired critical points that are difficult to anticipate and characterize. Indeed, visualizations (see Supplementary Figure 3) of the objective in low-dimensional cases confirm that the objective is geodesically nonconvex in general, containing several saddle points and local minima.

In such situations, finding a method that can reliably reach local minima becomes

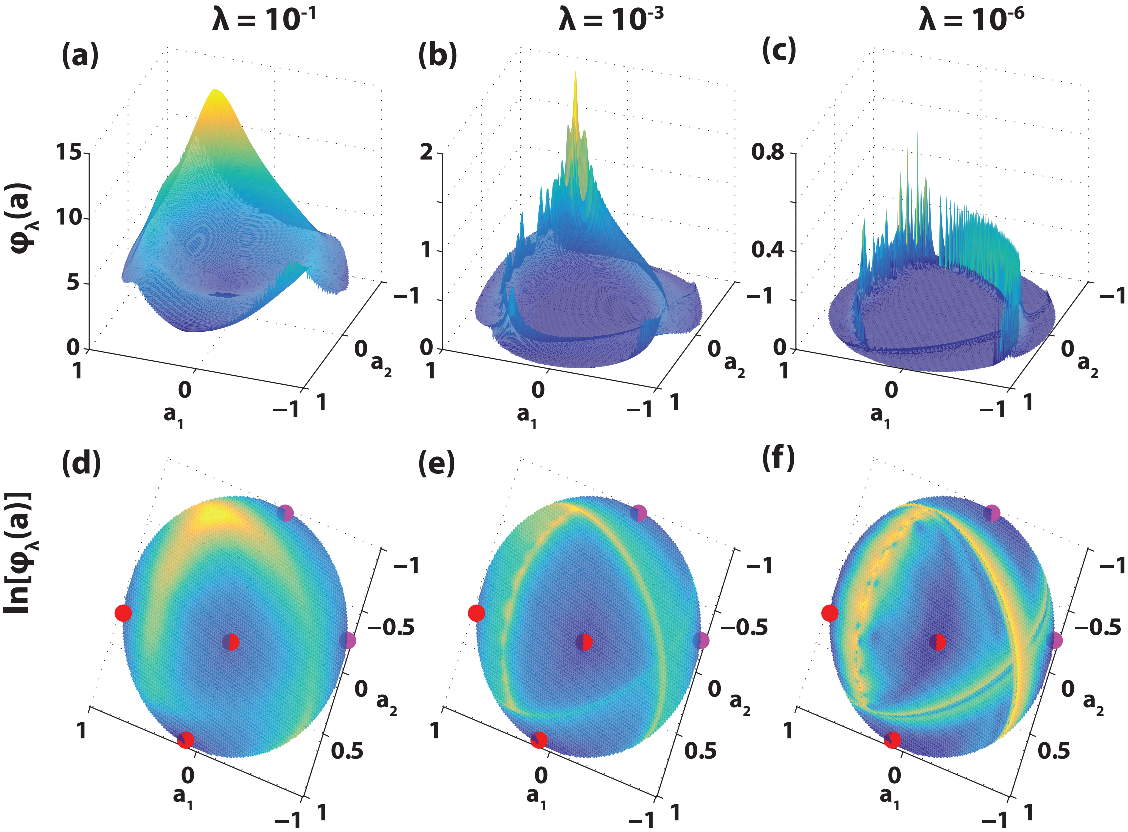

Supplementary Figure 3. Objective function geometry with varying values of λ. (a)-(c) The objective $\varphi_\lambda(\mathbf{a}) = \min_{\mathbf{x}} \frac{1}{2}\|\mathbf{a} * \mathbf{x} - \mathbf{a}_0 * \mathbf{x}_0\|_2^2 + \lambda\|\mathbf{x}\|_1$ over the hemisphere $\{(a_1, a_2) : a_1^2 + a_2^2 \le 1\}$ for $\lambda = 10^{-1}$, 10^{-3}, and 10^{-6}. Each pair (a_1, a_2) uniquely determines $\mathbf{a} \in \mathbb{S}^2$. The truth kernel is $\mathbf{a}_0 = \mathbb{P}_{\mathbb{S}^2}([1, 8, 2])$ (the vector $[1, 8, 2]$ projected onto the sphere) and entries of $\mathbf{x}_0 \in \mathbb{R}^{256}$ are drawn independently and randomly from a Bernoulli-Gaussian distribution with $x_i \overset{\text{i.i.d.}}{\sim} \text{Bern}(0.1) \cdot \mathcal{N}(0, 1)$. (d)-(f) Logarithm of the objective functions shown in (a)-(c), respectively. The truth kernel $\mathbf{a}_0$ and its shift-truncations $\mathbb{P}_{\mathbb{S}^2}([8, 2, 0])$, $\mathbb{P}_{\mathbb{S}^2}([0, 1, 8])$ are shown in red, and sign-flips $\mathbb{P}_{\mathbb{S}^2}(-[8, 2, 0])$, $\mathbb{P}_{\mathbb{S}^2}(-[0, 1, 8])$ are shown in magenta. Brighter colors on the surface plots indicate higher objective value. Notice that each signed shift-truncation shown on the hemisphere is close to a corresponding local minimum. Furthermore, the objective landscape becomes less regularized as λ shrinks, with many local minima appearing for $\lambda = 10^{-6}$.

more involved. For the present study we employ the Riemannian Trust-Region Method (RTRM) [23]. The standard TRM produces updates for iterates in Euclidean space by minimizing a quadratic approximation of the objective within a neighborhood of the iterate. The neighborhood radius is maintained at each iteration to limit the deviation of the quadratic approximation from the true objective. This procedure results in a second-order method guaranteeing that a local minimum will be attained so long as the stationary points of the

objective are nondegenerate. The RTRM extends this method to Riemannian submanifolds embedded in Euclidean spaces – such as the sphere $\mathcal{S}$ – by augmenting TRM operations to become consistent with the manifold geometry. Consequently, the RTRM provides strong guarantees that a local minimum of the objective will be attained over $\mathcal{S}$.

Other descent methods can potentially be extended to find local minima in a manifold setting. Except for pathological examples, solving problems on Euclidean spaces using gradient descent with random initializations will converge to a local minimum almost surely [24], and noisy gradient descent is guaranteed to efficiently converge to a local minimum [25]. As a second-order method, TRM enjoys significantly faster convergence with regards to iterations but with increased computation per iteration, as well as better overall tail convergence (when iterates are close to local minima).

Solving the SBD-STM Problem with RTRM

Smoothing the Sparse Regularizer:

Although we would like to solve problemSupplementary Equation (22) using RTRM, the presence of the ℓ_1 regularizer forbids second-order information to be extracted from $\varphi_\lambda(\mathcal{A}) = \min_{\mathcal{X}} \psi_\lambda(\mathcal{X}, \mathcal{A}) = \min_{\mathcal{X}} \frac{1}{2}\|\mathcal{A} \circledast \mathcal{X} - \mathcal{Y}\|_F^2 + \lambda \cdot r(\mathcal{X})$. Since the variable $\mathcal{X}$ is marginalized by solving the convex problem

$$\mathcal{X}_*(\mathcal{A}; \lambda) \leftarrow \min_{\mathcal{X}} \frac{1}{2}\|\mathcal{A} \circledast \mathcal{X} - \mathcal{Y}\|_F^2 + \lambda \cdot r(\mathcal{X}) \tag{23}$$

dependent on $\mathcal{A}$, computing the Hessian $\nabla^2_{\mathcal{A},\mathcal{A}}\varphi_\lambda(\mathcal{A})$ requires access to $\nabla^2_{\mathcal{X},\mathcal{X}}\psi_\lambda(\mathcal{A}, \mathcal{X})$, which does not exist when ℓ_1-norm is present. Instead, a regularizer that enjoys the existence of second derivatives should be chosen to approximate the ℓ_1-norm. For this problem, we choose the *pseudo-Huber* regularizer $r_\mu(\mathcal{X})$, so that:

$$r(\mathcal{X}) = r_\mu(\mathcal{X}) \equiv \sum_{i,j} \mu^2 \left(\sqrt{1 + \mu^{-2} \cdot \mathcal{X}_{i,j}} - 1 \right), \tag{24}$$

where μ would typically be a small positive scalar ($\mu = 10^{-6}$ is chosen in experiments). We see that although $\lim_{\mu \to 0} r(\cdot) = \|\cdot\|_1$, this function has continuous derivatives of all orders.

Using a smoothed regularizer also helps to avoid troublesome high-order critical points. Although the ℓ_1-norm is a popular regularizer for producing sparse solutions, it can be problematic in the context of bilinear inverse problems. When $\mathcal{A}$ is far from $\pm\mathcal{A}_0$ on the sphere, solving $\mathcal{X}_* \leftarrow \min_{\mathcal{X}} \frac{1}{2}\|\mathcal{A} \circledast \mathcal{X} - \mathcal{Y}\|_2^2 + \lambda\|\mathcal{X}\|_1$ encourages entries of $\mathcal{X}$ to be exactly zero. If the entire coefficient map $\mathcal{X}$ is zero, then the gradient of φ with respect to $\mathcal{A}$ is also

zero, and descent methods make no progress. Employing a regularizer that encourages a few elements of $\mathcal{X}_*$ to be larg, while keeping other elements small (or "approximately sparse") circumvents this problem as the gradient is unlikely vanish.

Modified minimization problem. Based on the discussion above, we solve the following problem rather thanSupplementary Equation (22):

$$(\hat{\mathcal{A}}, \hat{\mathcal{X}}) \leftarrow \min_{\mathcal{A}\in\mathcal{S}} \left\{ \varphi_\lambda(\mathcal{A}) \equiv \min_{\mathcal{X}} \left[\psi_\lambda(\mathcal{A}, \mathcal{X}) \equiv \frac{1}{2}\|\mathcal{A} \circledast \mathcal{X} - \mathcal{Y}\|_F^2 + \lambda \cdot r(\mathcal{X}) \right] \right\} \tag{25}$$

which is the objective discussed in equation (4) of the main text.

Implementation of RTRM: The MATLAB package ManOpt [26] is used solveSupplementary Equation (25) using RTRM. To produce a quadratic approximation of the objective, one needs the ability to compute $\varphi_\lambda(\mathcal{A})$, $\nabla_\mathcal{A}\varphi_\lambda(\mathcal{A})$ as well as $\nabla^2_{\mathcal{A},\mathcal{A}}\varphi_\lambda(\mathcal{A})$ for any $\mathcal{A} \in \mathcal{S}$. By rewriting the convolution operator $\circledast$ in terms of *cyclic convolutions*, compact expressions can be derived for both the (Euclidean) gradient and Hessian, which are then mapped to their Riemannian equivalents via tools from the package. As an additional benefit, expression in terms of cyclic convolutions allows fast computation of $\circledast$ via the FFT.

As discussed earlier, the ℓ_1 regularizer is replaced with the pseudo-Huber norm to make second-order information available to the RTRM. The pseudo-Huber regularizer allows the precise solution ofSupplementary Equation (23) to be found using the algorithm proposed by Fountoulakis and Gondzio [27], which reports good performance when the observations are generated from a poorly conditioned linear system. Since the individual voltage slices of $\mathcal{A}$ are expected to be smooth and low-pass in the Fourier domain, the case where $(\mathcal{A} \circledast \cdot)$ is poorly conditioned is of practical interest.

The availability of a method to compute φ_λ and its Riemannian gradient and Hessian allows one to solveSupplementary Equation (25) using RTRM. We denote this procedure using the notation

$$\hat{\mathcal{A}} \leftarrow \texttt{ASolve}\big(\mathcal{A}_{\text{init}}, \lambda; \mathcal{Y}, (m_1, m_2)\big),$$

passing the regularization parameter λ and an initialization $\mathcal{A}_{\text{init}}$ for $\mathcal{A}$ as input arguments. The dependence of `ASolve` on the observation $\mathcal{Y}$ and the kernel size (m_1, m_2) (which is implicitly provided by $\mathcal{A}_{\text{init}}$) is explicitly made, although we will write $\texttt{ASolve}\big(\mathcal{A}_{\text{init}}, \lambda\big)$ for brevity when the observation and kernel sizes are clear by context.

Poor estimates of $\hat{\mathcal{A}}$ can occur due to overfitting the noise term in the observation $\mathcal{Y}$. Higher values of λ help retain the quality of $\hat{\mathcal{A}}$ by enforcing sparsity in $\hat{\mathcal{X}}$. On the other hand,

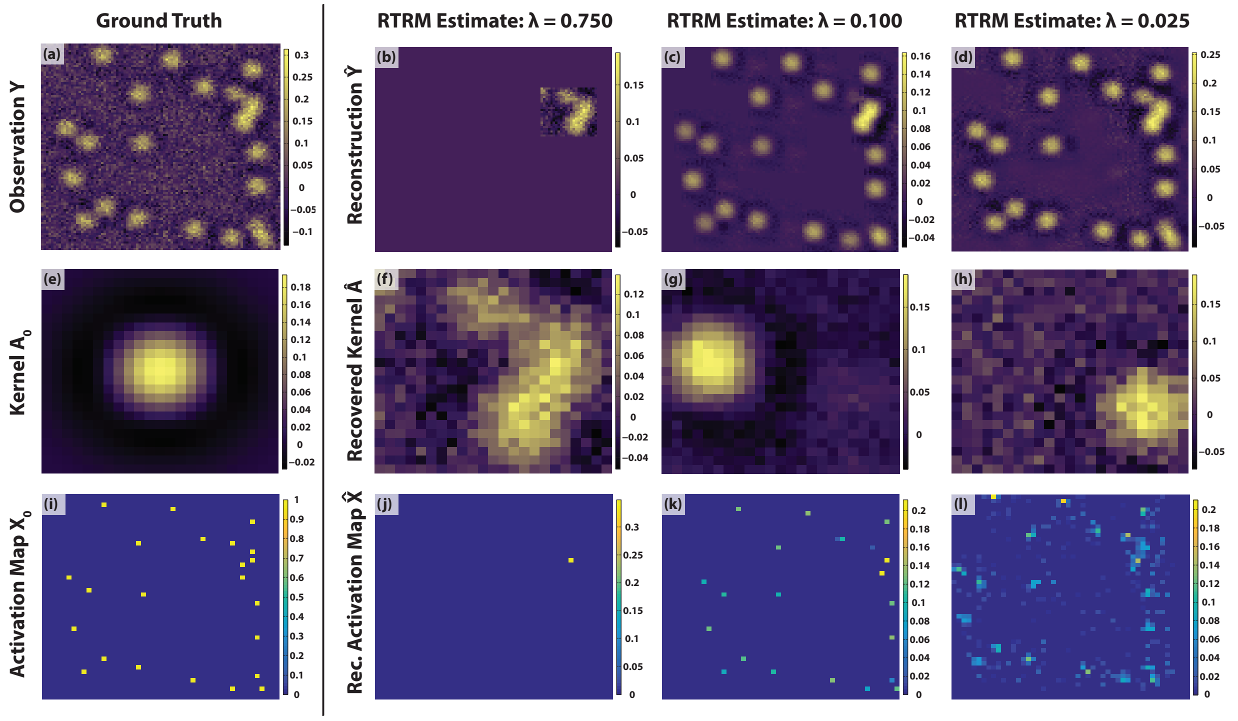

Supplementary Figure 4. RTRM estimates (without refinement) from a simulated observation $\mathcal{Y} = \mathcal{A} * \mathcal{X} + \mathcal{Z}$, with moderate noise $(\mathcal{Z})_{ij} \sim \mathcal{N}(0, 10^{-3})$ and using selected values of $\lambda = 0.750$, 0.100, and 0.025. The original observation $\mathcal{Y}$ is shown in (a), which was determined through the convolution of the truth kernel $\mathcal{A}_0$ and truth activation map $\mathcal{X}_0$, depicted in (e) and (i), respectively. Reconstructed observations from the convolution $\hat{\mathcal{Y}} = \hat{\mathcal{A}} * \hat{\mathcal{X}}$ for various values of λ are shown in (b)-(d). Observe that recovered kernels (f)-(h) $\hat{\mathcal{A}}$ correspond to shift-truncations of the truth kernel $\mathcal{A}_0$ shown in (e), but the low-energy side lobes tend to be lost under noise. Recovered activation locations from $\hat{\mathcal{X}}$ in (j)-(l) correspond to shift-truncations of the ground truth $\mathcal{X}_0$ in (i), but are not exact – recovery is particularly poor for nearby activation locations. Recovery in terms of the locations and relative magnitudes in $\hat{\mathcal{X}}$, as well as the details of $\hat{\mathcal{A}}$, is best when λ is chosen carefully. Setting λ too large causes $\hat{\mathcal{X}}$ to shrink excessively; conversely spurious activation locations are identified when λ is too low. Both situations degrade the quality of the estimate $\hat{\mathcal{A}}$.

if $\hat{\mathcal{X}}$ becomes too sparse (λ is too large), one can fail to recover activation locations from $\mathcal{X}_0$, meaning that there are fewer defects from the $\mathcal{Y}$ being taken into account by `ASolve`. This can also lead to poor estimates of $\mathcal{A}$.

Empirically, `ASolve` appears to perform well in simulations in which observations are generated by ground truths $\mathcal{A}_0$ and $\mathcal{X}_0$, and the entries of $\mathcal{Z}$ are drawn independently from the Gaussian distribution $\mathcal{N}(0, \eta)$. In particular, if the noise power η is not too high and $\mathcal{X}_0$ is sufficiently sparse, then a range of $\lambda \geq 0$ will lead $\hat{\mathcal{A}}$ to approximate *shift-truncations* of $\mathcal{A}_0$ regardless of the initialization to `ASolve`. This is demonstrated in Supplementary Figure 4.

The Complete SBD-STM Procedure: To address the issues of overfitting and shift-truncation, the complete SBD-STM procedure (Algorithm 1) refines the initial estimate produced by setting $\lambda = \lambda_0$,

$$\mathcal{A}_*^{(0)} \leftarrow \texttt{ASolve}\big(\mathcal{A}_{\text{init}}, \lambda_0\big),$$

through iterations of `ASolve` over a larger size of $\mathcal{A}$ and a decreasing sequence λ_k of regularization parameters until $\lambda_k \leq \lambda_{\text{end}}$ – this is often referred to as a *graduated optimization* or *continuation* procedure.

To deal with shift-truncations, the kernel size is enlarged to $m_1' \times m_2'$ in the refinement phase once the initial estimate $\mathcal{A}_*^{(0)}$ is obtained. At the end of each refinement, the variables are shifted so that the $m_1 \times m_2$ submatrix of $\mathcal{A}_*^{(k)}$ with the largest signal energy is centered. The choice of m_1' and m_2' depends on how far one expects the $\mathcal{A}_*^{(0)}$ to be shifted from the ground truth $\mathcal{A}_0$, but it cannot be too large to prevent the refinements $\mathcal{A}_*^{(k)}$ from converging into a different defect signature far larger than $m_1 \times m_2$ in size. In simulated experiments, we choose $m_i' = m_i + 2\left\lfloor \frac{m_i}{2} \right\rfloor$ for $i = 1, 2$, as it is unlikely a shift-truncation by any more than $\frac{m_i}{2}$ pixels can be a local minimum of φ_{λ_0}.

Regarding graduated continuation, we suggest that λ_0 be chosen relatively large (around 0.1 - 0.5) based our discussion in Section . This encourages a sparse $\mathcal{X}_*(\mathcal{A}; \lambda)$, and forces the activations to considerably favor regions of the image with prominent defect signatures. Although $\mathcal{A}_*^{(0)}$ will be affected by heavy bias and noise (see Supplementary Figure 4), this can be refined in later iterations.

Starting with $\lambda_1 = \lambda_0$, a geometrically decreasing sequence for $\{\lambda_k\}$ is chosen, i.e. $\lambda_k = \alpha \cdot \lambda_{k-1}$ for $k = 2, 3, \ldots$. This leaves the user to consider the decay rate $\alpha \in [0, 1)$ and the terminating regularization parameter λ_{end}. Based on Ref. 9, we recommend that λ_{end} be chosen proportionately to $\sqrt{n_1 \cdot n_2 \cdot \eta}$, in which η denotes the additive noise variance. In particular, observe that in the limit as $\lambda \to 0$ and in the absence of noise,Supplementary Equation (25) becomes equivalent to the equally constrained problem

$$\begin{aligned} \min_{\mathcal{X}, \mathcal{A} \in \mathcal{S}} \quad & r(\mathcal{X}) \\ \text{s.t.} \quad & \mathcal{A} \circledast \mathcal{X} = \mathcal{Y}. \end{aligned}$$

When considering the decay rate α, a smaller choice of α means fewer refinements are needed. However, we would also like to decrease slowly enough so that $\varphi_{\lambda_{k+1}}$ is not "too different" from φ_{λ_k}, in the sense that we do not want $\mathcal{A}_*^{(k)}$ to jump to a wildly different, possibly

Algorithm 1 Complete SBD-STM Procedure

Input:

- Observation $\mathcal{Y} \in \mathbb{R}^{n_1 \times n_2 \times s}$,
- Kernel size (m_1, m_2),
- Initial $\lambda_0 \geq 0$, decay rate $\alpha \in [0, 1)$, and final $\lambda_{\text{end}} \geq 0$.

Initial phase:

1. Randomly initialize: $\mathcal{A}^{(0)} \in \mathcal{S} = \mathbb{S}^{m_1 \times m_2 \times s}$.
2. $\mathcal{A}_*^{(0)} \leftarrow \texttt{ASolve}\left(\mathcal{A}^{(0)}, \lambda_0\right)$.

Refinement phase:

1. Lifting: Get $\mathcal{A}^{(1)} \in S' = \mathbb{S}^{m_1' \times m_2' \times s}$ by zero-padding the edges of $\mathcal{A}_*^{(0)}$ with a border of width $\lfloor \frac{m_i}{2} \rfloor$.
2. Set $\lambda_1 = \lambda_0$.
3. Continuation: **Repeat** for $k = 1, 2, \ldots$ **until** $\lambda_k \leq \lambda_{\text{end}}$,
   (a) $\mathcal{A}_*^{(k)} \leftarrow \texttt{ASolve}\left(\mathcal{A}^{(k)}, \lambda_k\right)$,
   (b) Centering:
      i. Find the size $m_1 \times m_2$ submatrix of $\mathcal{A}_*^{(k)}$ that maximizes the Frobenius (square) norm across all $m_1 \times m_2$ submatrices.
      ii. Get $\mathcal{A}^{(k+1)}$ by shifting $\mathcal{A}_*^{(k)}$ so that the chosen $m_1 \times m_2$ restriction is in the center, removing and zeropadding entries as needed.
      iii. Normalize $\mathcal{A}^{(k+1)}$ so it lies in $\mathcal{S}'$.
   (c) Set $\lambda_{k+1} = \alpha \lambda_k$.

Output:

1. Extract $\hat{\mathcal{A}} \in \mathcal{S}$ by extracting the restriction of the final $\mathcal{A}^{(k+1)}$ to the center $m_1 \times m_2$ window.
2. Find the corresponding activation map $\hat{\mathcal{X}} \in \mathbb{R}^{n_1 \times n_2}$ by solving $\min_{\mathcal{X}} \psi_{\lambda_k}\left(\hat{\mathcal{A}}, \mathcal{X}\right)$.

malign, local minimum when producing $\mathcal{A}_*^{(k+1)}$.

In our benchmarking experiments described in the main text, the varying noise level prohibits us from carefully tuning λ_{end}, but even without graduated continuation (single refinement, $\lambda_1 = \lambda_0 = 0.5$), the SBD-STM procedure produces qualitatively similar and

informative estimates of $\mathcal{A}_0$, albeit slightly suboptimal.

SUPPLEMENTARY NOTE 4: Application to Image Deblurring

In this section, we present an extension of our algorithmic approach that addresses an image deblurring problem in computer vision. Image deblurring aims to recover a sharp natural image from its blurred observation due to some unknown photographic process, such as a shaking camera or defocusing [28–30]. Although natural images are generally not sparse, it is widely acknowledged that sparsity exists in its spatial gradient [28, 31].

Suppose $\mathbf{Y} = \mathbf{A}_0 * \mathbf{X}_0$ is the observed blurry image, which is represented as the convolution of the original sharp image $\mathbf{X}_0$ and a kernel $\mathbf{A}_0$ that models the blurring. Owing to the linearity of the convolution operator, the gradient of the observed blurred image must equal the convolution of the blurring kernel $\mathbf{A}_0$ and the gradient of the original sharp image, which possesses the requisite sparsity needed in our algorithm. For instance, we have in two dimensions:

$$\nabla_x \mathbf{Y} = \mathbf{A}_0 * \nabla_x \mathbf{X}_0$$

$$\nabla_y \mathbf{Y} = \mathbf{A}_0 * \nabla_y \mathbf{X}_0$$

where ∇_x and ∇_y denote derivatives in the x and y directions, respectively. Since the gradients $\nabla_x \mathbf{X}_0$ and $\nabla_y \mathbf{X}_0$ are sparse signals by hypothesis, the image deblurring SBD problem can be cast as the following optimization problem:

$$\min_{\mathbf{A}\in\mathbb{S}_*,\mathbf{X}_x,\mathbf{X}_y} \left[\frac{1}{2}\|\nabla_x \mathbf{Y} - \mathbf{A} * \mathbf{X}_x\|_F^2 + \lambda \cdot r(\mathbf{X}_x) + \frac{1}{2}\|\nabla_y \mathbf{Y} - \mathbf{A} * \mathbf{X}_y\|_F^2 + \lambda \cdot r(\mathbf{X}_y)\right] \quad (26)$$

where $\mathbb{S}_*$ denotes the intersection of the unit sphere and the positive orthant. The optimization variables $\mathbf{X}_x$ and $\mathbf{X}_y$ correspond to the horizontal and vertical gradients of $\mathbf{X}$, respectively.

The non-negativity of the blurring kernel $\mathbf{A}$ removes sign ambiguity during the recovery process, contrasted to the STM problem described in Section . With minor modifications, the two-stage procedure used for SBD-STM can be applied to image deblurring to determine reliable estimates of the original sharp image. See Supplementary Figure 5 for a demonstration of the image deblurring process using this approach.

SUPPLEMENTARY NOTE 5: SBD-STM on High-Dimensional Simulated Measurements

Using the methods described in Section , we constructed artifical STM measurements of a material with 70 identical point defects distributed across a 50×50 atomic square lattice.

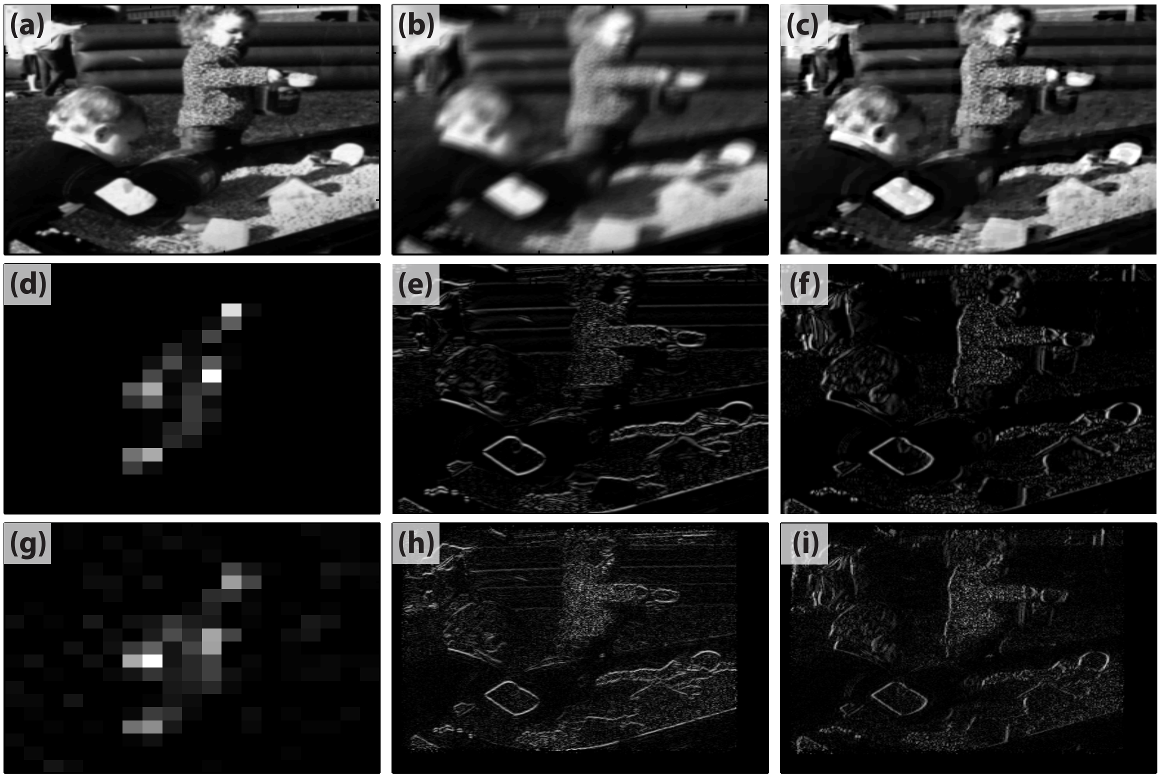

Supplementary Figure 5. Demonstration of image deblurring by solving the SBD problem in-Supplementary Equation (26). (a) Original sharp image $\mathbf{X}_0$ (b) Observed blurred image $\mathbf{Y}$ (c) Recovered deblurred image $\hat{\mathbf{X}}$ (d) Ground truth of blurring kernel $\mathbf{A}_0$ (e) Horizontal gradient of original image $\nabla_x \mathbf{X}_0$ (f) Vertical gradient of original image $\nabla_y \mathbf{X}_0$ (g) Recovered blurring kernel $\hat{\mathbf{A}}$ (h) Recovered horizontal gradient $\hat{\mathbf{X}}_x = \nabla_x \hat{\mathbf{X}}$ (i) Recovered vertical gradient $\hat{\mathbf{X}}_y = \nabla_y \hat{\mathbf{X}}$.

As discussed in the main text, the convolutional data model for STM measurements has the general form:

$$\mathcal{Y} = \mathcal{A} \boxast \mathcal{X} + \mathcal{Z}$$

The simulated dataset consisted of 41 constant-bias scans of the same 256×256 pixel measurement grid and included additive zero-mean Gaussian noise. Supplementary Figure 6 displays the algorithm outputs on two separate slices of simulated data.

The Re-FTs of the raw observations $\mathcal{Y}$ show some faint structure, which is obscured by phase noise associated with random defect locations. By using SBD-STM, one deconvolves the noisy measurement $\mathcal{Y}$ to recover the underlying kernel $\hat{\mathcal{A}}$ and the activation map $\hat{\mathcal{X}}$ marking the locations of the defects. As shown on the rightmost column of Supplementary Figure 6 the corresponding FTs of the recovered $\hat{\mathcal{A}}$ are uncontaminated by the phase noise inherent in the FT of $\mathcal{Y}$, leaving behind the "true" structure of the defect. The strong peaks

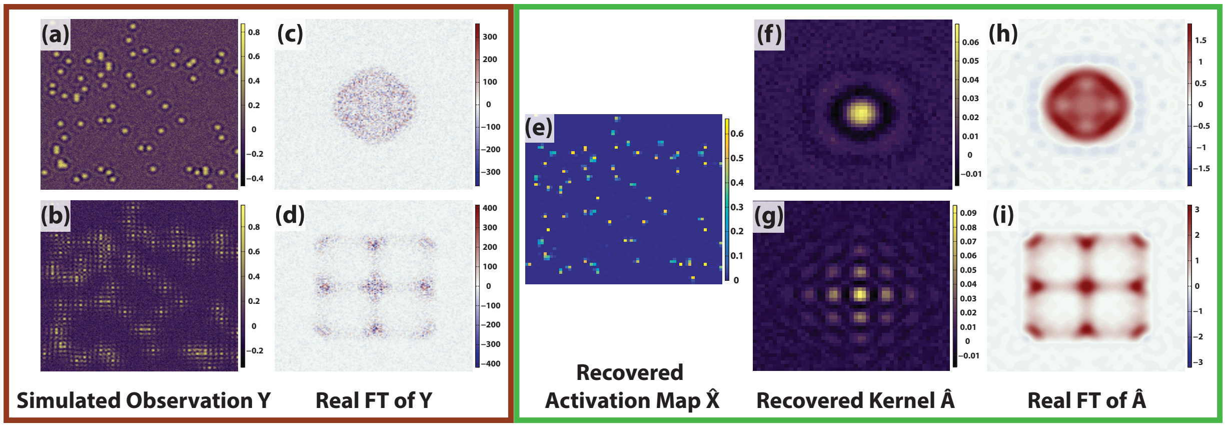

Supplementary Figure 6. SBD-STM results on simulated noisy STM data with 70 localized defects. Results for the FT-STM and SBD-STM methodologies on the same dataset are enclosed in the red and green boxes, respectively. (a)-(b) Two separate constant-bias slices from the same $\mathcal{Y} \in \mathbb{R}^{256\times256\times41}$ dataset (c)-(d) The corresponding Re-FT of the simulated observations in (a)-(b), respectively. Shown in the green box are SBD-STM results: (e) the recovered activation map $\hat{\mathcal{X}}$ (globally refined across all 41 bias slices) and (f)-(g) the recovered kernel $\hat{\mathcal{A}}$ for each constant-bias slice of the observed $\mathcal{Y}$ from (a)-(b). (h)-(i) The corresponding Re-FTs of each recovered kernel $\hat{\mathcal{A}}$. There is significant improvement in data fidelity in the SBD-STM results in (h)-(i) compared to results from the standard FT-STM methodology in (c)-(d). All Re-FT spectra are shown with $-3\pi/5a \le q_x, q_y \le 3\pi/5a$.

Re-FT spectrum present in these images are consistent with the allowed elastic scatterings of the underlying square lattice. Such observations are less evident from Re-FTs of the original $\mathcal{Y}$ since phase noise suppresses the strong peaks.

SUPPLEMENTARY NOTE 6: Benchmarking the SBD-STM Approach

We use the artificial STM data in the previous section to generate measurements $\mathcal{Y}$ for assessing the SBD-STM's ability to provide robust estimates of $\mathcal{A}_0$ and $\mathcal{X}_0$ as various measurement parameters are varied. Parameters of interest include the measurement size $n \equiv n_1 \times n_2$, kernel size $m \equiv m_1 \times m_2$, kernel concentration θ, and additive noise variance η. To demonstrate the broad applicability of SBD-STM to multiple modalities of microscopy, we restrict our initial benchmarking datasets to single-energy measurements $\mathcal{Y}_{\theta,\eta} \in \mathbb{R}^{n_1\times n_2}$. Under the convolutional data model, each observation is generated through the process:

$$\mathcal{Y}_{\theta,\eta} = \tilde{\mathcal{A}}_0 \circledast \mathcal{X}_\theta + \mathcal{Z}_\eta \tag{27}$$

where $\tilde{\mathcal{A}}_0 \in \mathbb{R}^{n_1\times n_2}$ is the zero-padded extension of the truth kernel $\mathcal{A}_0 \in \mathbb{R}^{m_1\times m_2}$, which is chosen from TB simulation results described in Section . The stochastic contributions for each measurement $\mathcal{Y}_{\theta,\eta}$ are contained in the random activation map $\mathcal{X}_\theta \in \mathbb{R}^{m_1\times m_2}$ as

a Bernoulli process with parameter θ and in $\mathcal{Z}_\eta \in \mathbb{R}^{m_1 \times m_2}$ as zero-mean Gaussian noise process with variance η.

A series of independent measurements is produced followingSupplementary Equation (27) for several candidate values of θ and η. Each artificial measurement $\mathcal{Y}_{\theta,\eta}$ is processed by SBD-STM, yielding estimates $\hat{\mathcal{A}}_{\theta,\eta}$ and $\hat{\mathcal{X}}_{\theta,\eta}$ of $\hat{\mathcal{A}}_0$ and $\hat{\mathcal{X}}_\theta$, respectively. To reduce experimental ambiguity, the SBD-STM regularizer parameter λ is fixed at 0.5 for these benchmarking trials. In practice, λ can be appropriately adjusted to suit specific measurement and model parameters, leading to results that are generally favorable to SBD-STM outputs with fixed λ. As described in the main text, we assess the quality of SBD-STM kernel recovery by defining the real-space error metric:

$$\epsilon(\hat{\mathcal{A}}_{\theta,\eta}, \mathcal{A}_0) \equiv \frac{2}{\pi} \arccos \left| \langle \hat{\mathcal{A}}_{\theta,\eta}, \mathcal{A}_0 \rangle \right|$$

where $\langle \hat{\mathcal{A}}_{\theta,\eta}, \mathcal{A}_0 \rangle \in [-1, 1]$ denotes the inner product between vectorizations of $\hat{\mathcal{A}}_{\theta,\eta}$ and $\mathcal{A}_0$. The mean of $\epsilon(\hat{\mathcal{A}}_{\theta,\eta}, \mathcal{A}_0)$ is the average angle (normalized by $\frac{\pi}{2}$) between the two vectors on the hemisphere. Thus this metric provides an indication of the overall SBD-STM recovery performance for measurements with fixed θ and η while the spread of $\epsilon(\hat{\mathcal{A}}_{\theta,\eta}, \mathcal{A}_0)$ reveals the stability of SBD-STM to stochastic fluctuations ascribed to θ and η.

Real Space Error vs Kernel Concentration and High-Amplitude Noise

To explore the evolution of $\epsilon(\hat{\mathcal{A}}_{\theta,\eta}, \mathcal{A}_0)$ on the kernel concentration and a wider range of additive noise variances than in the main text, additional collections of measurements $\mathcal{Y}_{\theta,\eta}$ of fixed size $n = 185 \times 185$ were constructed fromSupplementary Equation (27) with $\mathcal{A}_0$ of fixed size $m = 35 \times 35$. 20 independent measurements were made for each ordered pair (θ, η), with η ranging from 0 to 0.01 and using the same θ values in Figure 4(b) of the main text. A summary of the real-space recovery errors for these trials is shown in Supplementary Figure 7. Supplementary Figure 8 shows representative observations $\mathcal{Y}_{\theta,\eta}$ and the corresponding recovered kernel $\hat{\mathcal{A}}_{\theta,\eta}$ for selected SNR and θ values in Supplementary Figure 7.

Fourier Space Error Comparison

Supplementary Figure 9 displays the Real Part of the FTs (Re-FTs) of the real-space results presented in Supplementary Figure 8. From these examples, one observes that the Fourier-space errors from raw measurements $\mathcal{Y}_{\theta,\eta}$ gradually improve with increasing concentration. However, the presence of phase noise and experimental noise washes away the

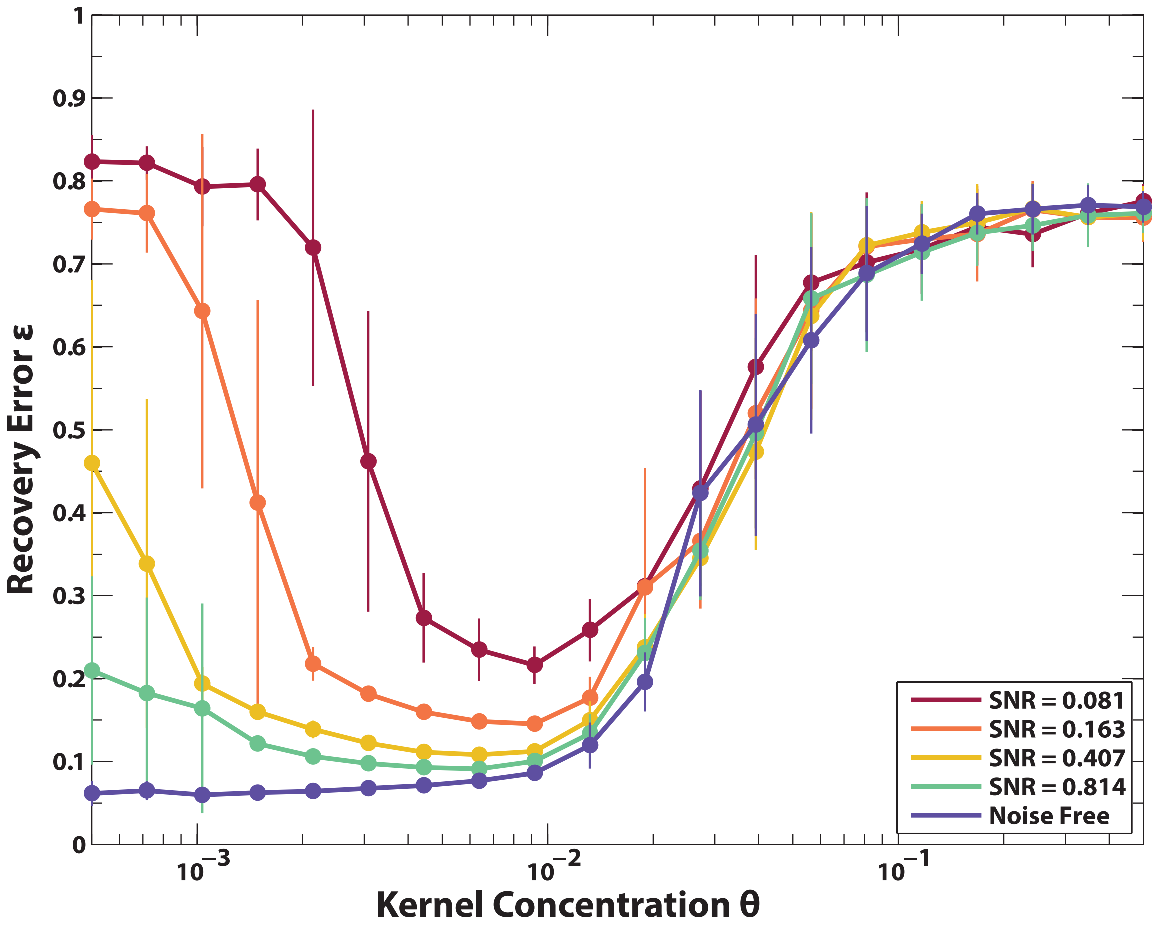

Supplementary Figure 7. Averaged real-space errors of the recovered kernel $\hat{\mathcal{A}}_{\theta,\eta} \in \mathbb{R}^{35\times35}$ against the truth kernel $\mathcal{A}_0 \in \mathbb{R}^{35\times35}$ in 20 independent simulated STM measurements $\mathcal{Y}_{\theta,\eta} \in \mathbb{R}^{185\times185}$. Solid lines indicate the kernel recovery error $\epsilon(\hat{\mathcal{A}}_{\theta,\eta}, \mathcal{A}_0)$ vs. kernel concentration θ in the presence of additive Gaussian noise with variance η. Examples of simulated measurements in this figure and their corresponding SBD-STM recovered kernels are shown in Supplementary Figure 8.

detailed structure found in the FT of the truth kernel. The corresponding Fourier-space errors from $\hat{\mathcal{A}}$ exhibit similar trends as the real-space errors with increasing concentration. There is an improvement in the FT errors as the kernel concentration is increased to a range spanning $0.001 \lesssim \theta \lesssim 0.01$. However, even in the high concentration limit (right-most column) the Re-FT of $\hat{\mathcal{A}}$ possesses many detailed features present in the truth kernel Re-FT that are absent in the Re-FT of $\mathcal{Y}_{\theta,\eta}$.

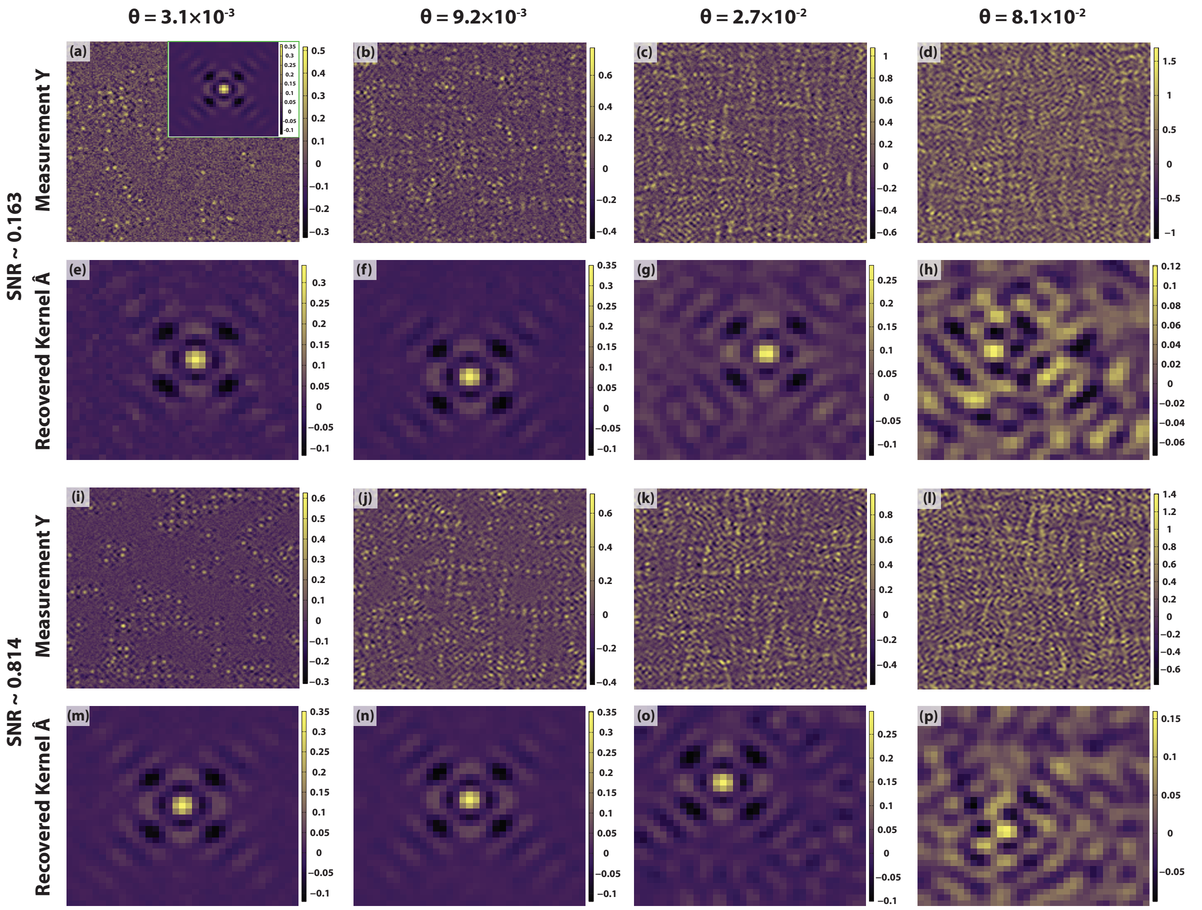

Supplementary Figure 8. (a)-(p) Example measurements and recovered kernels for select kernel concentrations and SNRs from Supplementary Figure 7. The truth defect pattern $\mathcal{A}_0$ for these measurements is shown in the inset of (a). SBD-STM is able to recover a reasonable estimate of the defect kernel $\hat{\mathcal{A}}$, even with poor SNR ≈ 0.163 that prohibits reliable deconvolution by inspection. These kernel estimates also include detailed interference patterns surrounding the bright peak in the LDoS, which are visually obscured in $\mathcal{Y}$ by the high noise levels.

SUPPLEMENTARY NOTE 7: Drift Correction

All of the experimental STM spectroscopy maps shown in figures 1, 5 and 6 of the main text have been corrected for drift and piezo errors. The acquisition time for these spectroscopy maps ranges from 24-48 hours, over the course of which we typically observe a few nanometers of drift. Due to the large spatial sizes of these images, atomic resolution is not maintained at the pixel counts (256 pixels on side) for data acquisition, and the atomic positions cannot be used for drift correction. Instead, fast ($\approx$ 15 minute) topographic scans are obtained for reference on the surface of the crystal before and after the slow spectroscopic imaging. Shown in Supplementary Figure 10 is the topograph obtained during the spectroscopic imaging, together with a reference scan obtained after the spectroscopic

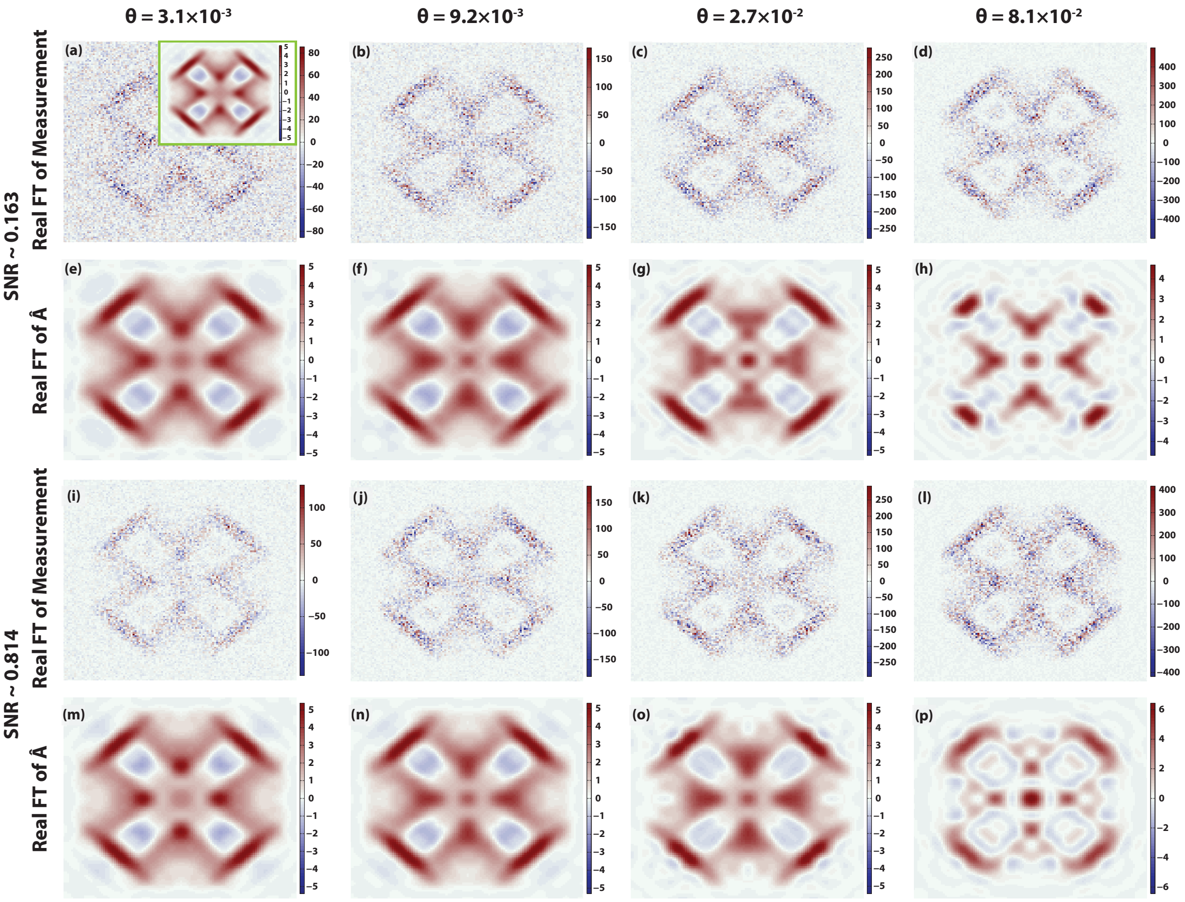

Supplementary Figure 9. (a)-(p) Re-FTs of the raw observations and recovered kernels from Supplementary Figure 8. All Re-FT spectra are shown with $-3\pi/5a \leq q_x, q_y \leq 3\pi/5a$. The Re-FT of the truth defect pattern $\mathcal{A}_0$ for these results is shown in the inset of (a). Phase-sensitive recovery of the scattering patterns is only observed in the Re-FT of recovered kernels. Note the difference in the colorbar scales between the Re-FT of the original measurements and recovered kernels.

imaging is performed. This topograph corresponds to the spectroscopic data shown in Figure 1a of the main text. Several vacancies on the sodium lattice on the top surface can be seen in both images. An affine transformation is used to match the positions of the vacancies in the topograph obtained during spectroscopic imaging to the reference scan, and this affine transformation is applied to the spectroscopic data to obtain drift corrected images. The fast reference scans themselves are calibrated on the herringbone reconstruction of Au(111) to remove piezo errors.

SUPPLEMENTARY NOTE 8: 2-fold Symmetrization

We show an example of a recovered kernel before and after symmetrization. The recovered kernel is the same as in Figure 5b) in the manuscript ($T = 71$ K, $I = 59$ pA, $V_{set} = -100$

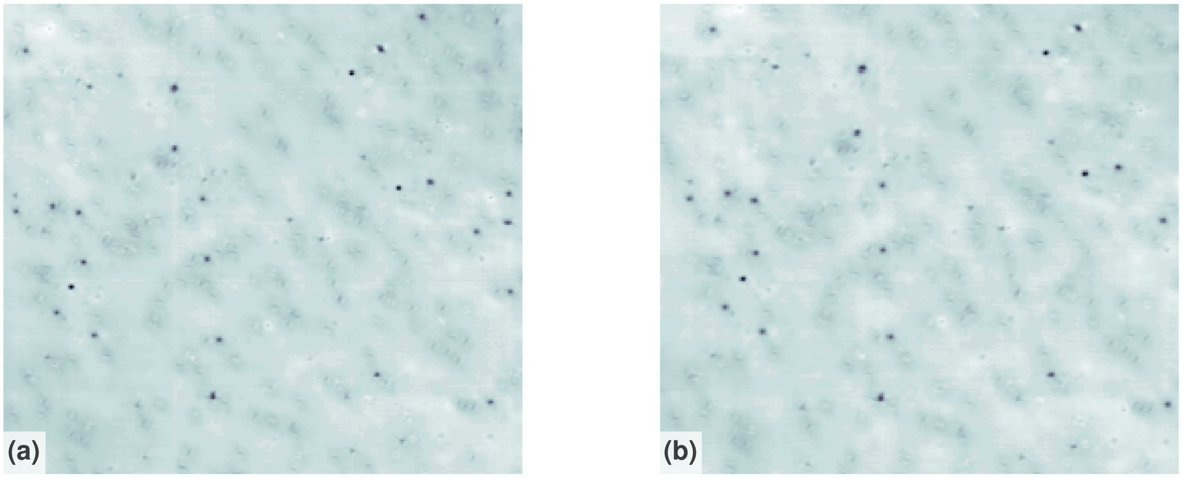

Supplementary Figure 10. (a) Topograph acquired during spectroscopic imaging, corresponding to the spectroscopic map shown in Figure 1a of the main text. Image acquisition time was 38 hours. (b) Reference topographic image obtained from a 20 minute scan after the spectroscopic map was completed. Both images show the presence of several sodium vacancies (black dots), and an affine transform is used to match the topograph in (a) to the one in (b). This affine transformation is then used on the spectroscopy images to correct for drift. The reference topograph in (b) itself was calibrated against the herringbone reconstruction of Au(111) to remove piezo errors.

mV, 114×114 nm).

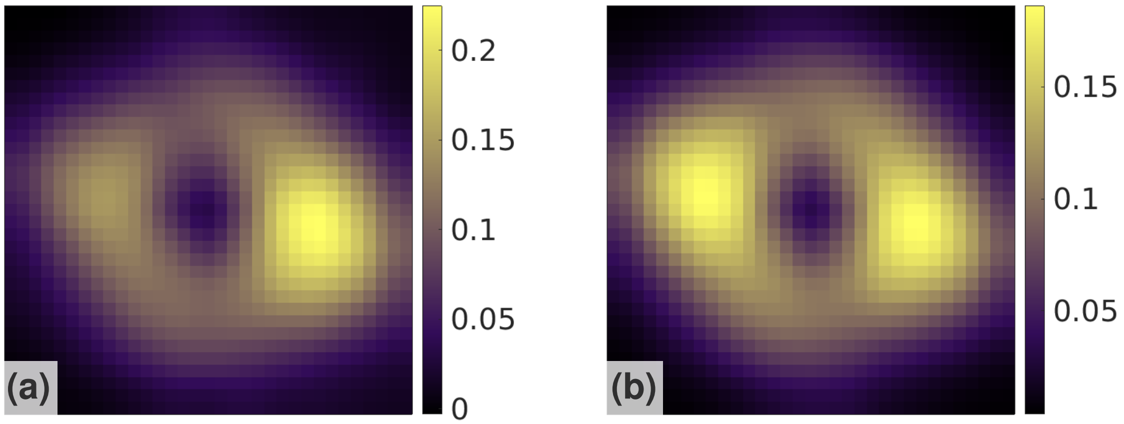

Supplementary Figure 11. A recovered kernel of high quality $NaFe_{1-x}Co_xAs$ a) before and b) after 2-fold rotational symmetrization. The experimental parameters were $T = 71$ K, $I = 59$ pA, $V_{set} = -100$ mV, 114×114 nm.

SUPPLEMENTARY NOTE 9: HAEM Calculation for Co-doped NaFeAs

To describe the expected results for the HAEM procedure for Co-doped NaFeAs, we employ a ten-orbital tight-binding parametrization of the electronic structure in NaFeAs[32] fitted to the available ARPES data[33]. The band structure is shown in Fig.12 and consists of the three hole pockets near the Γ-point of the first Brillouin zone and two electron pockets, centered near the M-point of the Brillouin Zone. Here we employ the notations of the two Fe-ions unit cell.

The multiple scattering of quasiparticles by a non-magnetic impurity was calculated as a correction to the local density of states (LDOS), using the standard T-matrix approach describing multiple scattering by a single impurity. For simplicity we consider multiple scattering from a single pointlike spherically symmetric non-magnetic impurity, i.e. the impurity potential $\hat{U}$ is taken to be well-localized and independent of spin. The t-matrix is then no longer momentum dependent, and is related to the impurity potential by $\hat{t} = \hat{U} + \hat{U}\hat{G}^0\hat{t}$. In particular, we compute the anti-symmetrized correction to the LDOS. In such

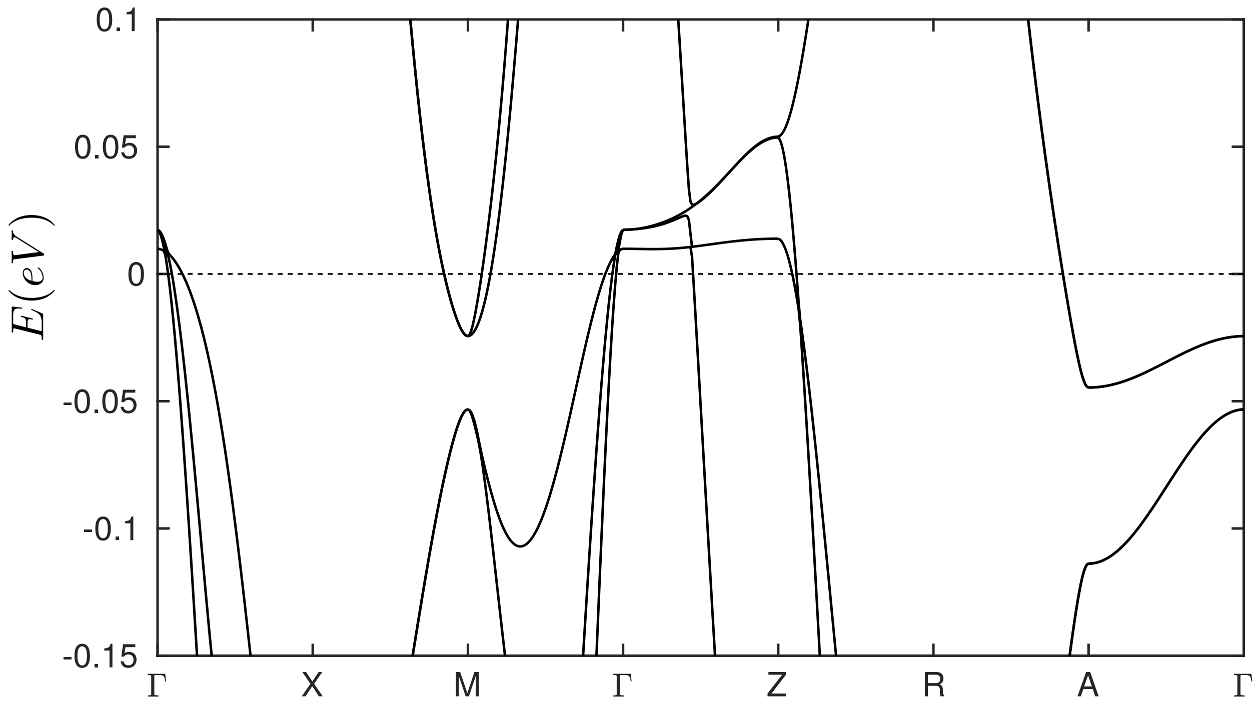

Supplementary Figure 12. Electronic band structure for the Co-doped NaFeAs along the high-symmetry point of the crystal, fitted to the ARPES data[33]

a case the solution for the t-matrix may be written,

$$\hat{t}(\omega) = [1 - \hat{U} \sum_{\mathbf{k}} \hat{G}(\mathbf{k}, \omega)]^{-1} \hat{U}. \tag{28}$$

Thus the position dependent correction to the LDOS reads may be written $\rho(\mathbf{q}, \omega) = \rho(\omega) + \delta\rho(\mathbf{q}, \omega)$ with

$$\delta\rho(\mathbf{q}, \omega) = -\frac{1}{\pi} \mathrm{Tr}\ \mathrm{Im} \sum_{\mathbf{k}} \hat{G}^0(\mathbf{k}, \omega) \hat{t}(\omega) \hat{G}^0(\mathbf{k} + \mathbf{q}, \omega) \tag{29}$$

where in the last equation we employ the analytic continuation to the advanced (retarded) Green's functions. Following previous analysis for LiFeAs[34] we assume the impurity potential to be diagonal in the orbital space with equal amplitudes for each orbital. For simplicity we considered weak impurity scatterer of 1meV for each of the orbitals.

Superconducting gap magnitudes on the electron and hole pockets were measured for Co-doped NaFeAs from ARPES[33, 35] yet their relative phases are unknown. In particular, the values of the superconducting gap on each band were taken to be $\Delta_h = 6.5$meV, $\Delta_e = 6.8$meV on the electron and the smaller hole pockets, respectively [35], and $\Delta_H = 3.5$meV on the larger hole pocket[33].

The antisymmetric part of the correction to the LDOS can be found as

$$\delta\rho^{-}(\mathbf{q},\omega) = \text{Tr Im}\sum_{\mathbf{k}} \tau_3 \hat{G}^0(\mathbf{k},\omega)\hat{t}(\omega)\hat{G}^0(\mathbf{k}+\mathbf{q},\omega), \tag{30}$$

where the t-matrix in the Nambu space is given by

$$\hat{t}(\omega) = \left[1 - \tau_3 \hat{U}\sum_{\mathbf{k}} \hat{G}(\mathbf{k},\omega)\right]^{-1} \tau_3 \hat{U} \tag{31}$$

and τ_3 is the corresponding component of the Pauli matrix.

Note, as was argued previously[34], the behavior of the total $\delta\rho^{-}(\omega)$ summed over all $\mathbf{q}$ wave vectors provides valuable information on whether the gap is a sign changing function itself without addressing the actual symmetry or detailed structure of the gap. This is essentially because the contributions arising from wave vectors that connect bands with order parameters of the same sign have negligible amplitude compared to those involving sign changes. The calculations within weak scattering limit shown in the main part of the paper consider the phases of the superconducting gap on the Fermi surface pockets corresponding to the s^{+-} and s^{++} structure of the gap. As one clearly sees there is a clear difference in the behavior of the $\delta\rho^{-}(\omega)$ for the sign-changing and the sign-preserving gaps. Most importantly, experimental results for $\hat{A}^{-}(\omega)$ are globally consistent with the sign changing gap.

SUPPLEMENTARY NOTE 10: Recovered Kernels for optimally doped $\mathbf{NaFe_{1-x}Co_xAs}$

Shown in figure 13 are recovered kernels at several energies from optimally doped $NaFe_{1-x}Co_xAs$. These kernels are used in the calculation of $\hat{\mathcal{A}}^{-}(\omega)$ in figure 6 of the main text.

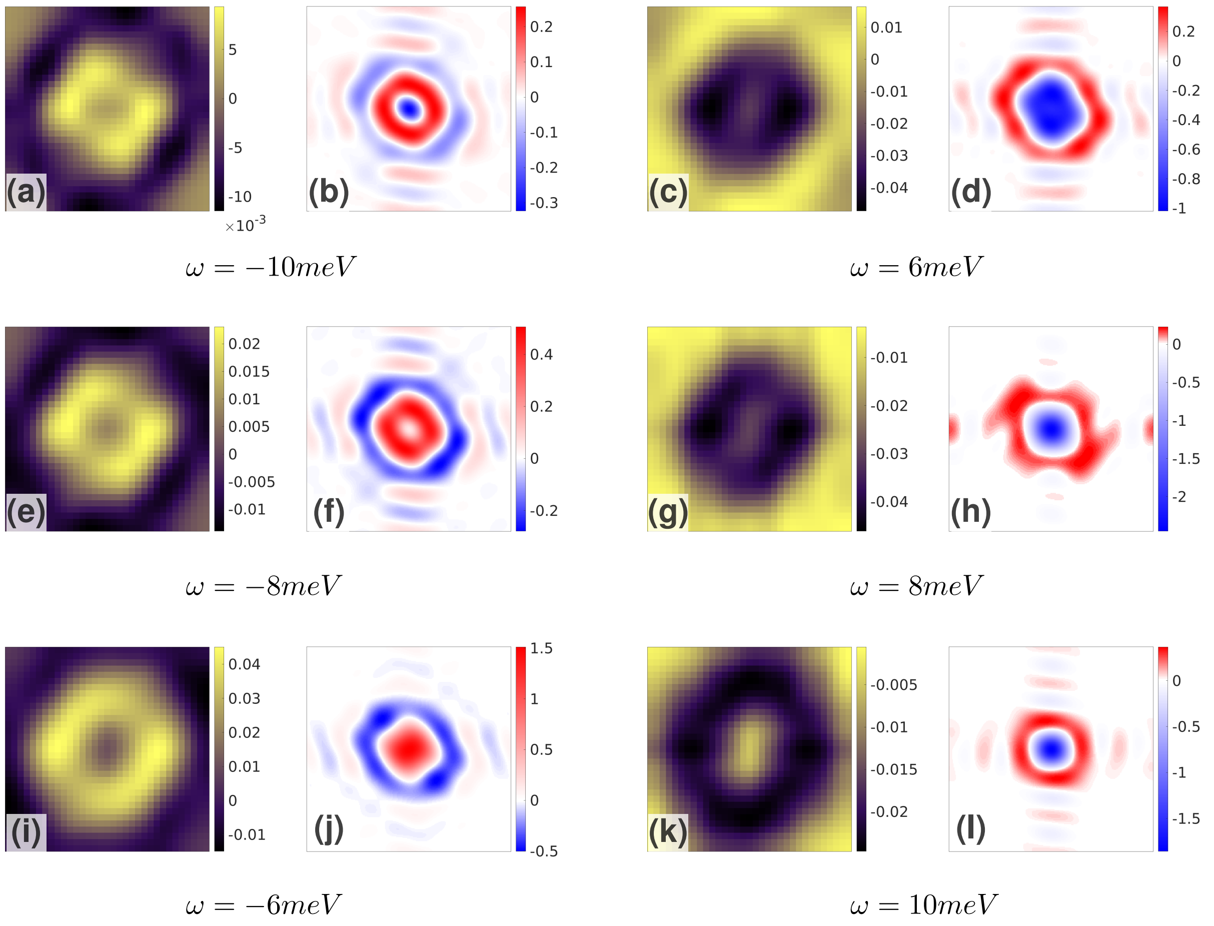

Supplementary Figure 13. Recovered real-space impurities (kernels) and their corresponding Re-FTs from $NaFe_{1-x}Co_xAs$ at optimal doping (x=0.02) at (a,b) -10 meV, (e,f) -8 meV, (i,j) -6 meV and (c,d) 6 meV, (g,h) 8 meV, and (k,l) 10 meV.

Supplementary References

[1] Neil W. Ashcroft and Nathaniel David Mermin, *Solid State Physics* (Cengage Learning, 1976).

[2] Michael Marder, *Condensed Matter Physics*, 2nd ed. (Wiley, 2010).

[3] E.N. Economou, *Green's Functions in Quantum Physics*, 3rd ed., Springer Series in Solid-State Sciences (Springer, 2006).

[4] C.J. Chen, *Introduction to Scanning Tunneling Microscopy*, Monographs on the Physics and Chemistry of Materials (Oxford University Press, 2008).

[5] David L Donoho and Michael Elad, "Optimally sparse representation in general (nonorthogonal) dictionaries via l1 minimization," Proceedings of the National Academy of Sciences **100**, 2197–2202 (2003).

[6] Emmanuel J Candes and Terence Tao, "Decoding by linear programming," IEEE transactions on information theory **51**, 4203–4215 (2005).

[7] Roman Vershynin, "Introduction to the non-asymptotic analysis of random matrices," arXiv preprint arXiv:1011.3027 (2010).

[8] Trevor Hastie, Robert Tibshirani, and Martin Wainwright, *Statistical learning with sparsity: the lasso and generalizations* (CRC Press, 2015).

[9] Martin J Wainwright, "Sharp thresholds for high-dimensional and noisy sparsity recovery using-constrained quadratic programming (lasso)," IEEE transactions on information theory **55**, 2183–2202 (2009).

[10] Emmanuel J Candes and Terence Tao, "Near-optimal signal recovery from random projections: Universal encoding strategies?" IEEE transactions on information theory **52**, 5406–5425 (2006).

[11] Emmanuel Candes and Justin Romberg, "Sparsity and incoherence in compressive sampling," Inverse problems **23**, 969 (2007).

[12] Simon Foucart and Holger Rauhut, *A mathematical introduction to compressive sensing*, Vol. 1 (Springer, 2013).

[13] Michael Lustig, David L Donoho, Juan M Santos, and John M Pauly, "Compressed sensing mri," IEEE Signal Processing Magazine **25**, 72–82 (2008).

[14] Alan V Oppenheim and Ronald W Schafer, *Discrete-time signal processing* (Pearson Higher Education, 2010).

[15] Ali Ahmed, Benjamin Recht, and Justin Romberg, "Blind deconvolution using convex programming," IEEE Transactions on Information Theory **60**, 1711–1732 (2014).

[16] Yanjun Li, Kiryung Lee, and Yoram Bresler, "Identifiability in blind deconvolution with subspace or sparsity constraints," IEEE Transactions on Information Theory **62**, 4266–4275 (2016).

[17] Yuejie Chi, "Guaranteed blind sparse spikes deconvolution via lifting and convex optimization," IEEE Journal of Selected Topics in Signal Processing **10**, 782–794 (2016).

[18] Emmanuel J Candès and Carlos Fernandez-Granda, "Towards a mathematical theory of super-

resolution," Communications on Pure and Applied Mathematics **67**, 906–956 (2014).

[19] Eftychios A Pnevmatikakis, Daniel Soudry, Yuanjun Gao, Timothy A Machado, Josh Merel, David Pfau, Thomas Reardon, Yu Mu, Clay Lacefield, Weijian Yang, *et al.*, "Simultaneous denoising, deconvolution, and demixing of calcium imaging data," Neuron **89**, 285–299 (2016).

[20] Anat Levin, Yair Weiss, Fredo Durand, and William T Freeman, "Understanding and evaluating blind deconvolution algorithms," in *2009 IEEE Conference on Computer Vision and Pattern Recognition* (2009) pp. 1964–1971.

[21] Subhasis Chaudhuri, Rajbabu Velmurugan, and Renu Rameshan, *Blind Image Deconvolution: Methods and Convergence*, 1st ed. (Springer International Publishing, 2014).

[22] Patrizio Campisi and Karen Egiazarian, *Blind image deconvolution: theory and applications* (CRC press, 2016).

[23] P-A Absil, Robert Mahony, and Rodolphe Sepulchre, *Optimization algorithms on matrix manifolds* (Princeton University Press, 2009).

[24] Jason D Lee, Max Simchowitz, Michael I Jordan, and Benjamin Recht, "Gradient descent converges to minimizers," University of California, Berkeley **1050**, 16 (2016).

[25] Rong Ge, Furong Huang, Chi Jin, and Yang Yuan, "Escaping from saddle pointsonline stochastic gradient for tensor decomposition," in *Proceedings of The 28th Conference on Learning Theory* (2015) pp. 797–842.

[26] N. Boumal, B. Mishra, P.-A. Absil, and R. Sepulchre, "Manopt, a Matlab toolbox for optimization on manifolds," Journal of Machine Learning Research **15**, 1455–1459 (2014).

[27] Kimon Fountoulakis and Jacek Gondzio, "A second-order method for strongly convex\ ell _1-regularization problems," Mathematical Programming **156**, 189–219 (2016).

[28] A. Levin, Y. Weiss, F. Durand, and W. Freeman, "Understanding blind deconvolution algorithms," IEEE Transactions on Pattern Analysis and Machine Intelligence **33**, 2354–2367 (2011).

[29] D. Kundur and D. Hatzinakos, "Blind image deconvolution," Signal Processing Magazine, IEEE **13**, 43–64 (1996).

[30] Dilip Krishnan and Rob Fergus, "Fast image deconvolution using hyper-laplacian priors," in *Advances in Neural Information Processing Systems 22*, edited by Y. Bengio, D. Schuurmans, J. D. Lafferty, C. K. I. Williams, and A. Culotta (Curran Associates, Inc., 2009) pp. 1033–1041.

[31] Rob Fergus, Barun Singh, Aaron Hertzmann, Sam T. Roweis, and William T. Freeman, "Removing camera shake from a single photograph," ACM Trans. Graph. **25**, 787–794 (2006).

[32] Helmut Eschrig and Klaus Koepernik, "Tight-binding models for the iron-based superconductors," Phys. Rev. B **80**, 104503 (2009).

[33] S. Thirupathaiah, D.V. Evtushinsky, J. Maletz, V.B. Zabolotnyy, A.A. Kordyuk, T.K. Kim, S. Wurmehl, M. Roslova, I. Morozov, B. Büchner, and S.V. Borisenko, "Weak-coupling superconductivity in electron-doped nafe0.95co0.05as revealed by arpes," Phys. Rev. B **86**, 214508 (2012).

[34] D. Altenfeld, P. J. Hirschfeld, I. I. Mazin, and I. Eremin, "Detecting sign-changing superconducting gap in lifeas using quasiparticle interference," Phys. Rev. B **97**, 054519 (2018).

[35] Z.-H. Liu, P. Richard, K. Nakayama, G.-F. Chen, J.-B. He S. Dong, D.-M. Wang, T.-L. Xia, K. Umezawa, T. Kawahara, S. Souma, T. Sato, T. Takahashi, T. Qian, Yaobo Huang, Nan Xu, Yingbo Shi, H. Ding, and S.-C. Wang, "Unconventional superconducting gap in nafe0.95co0.05as observed by angle-resolved photoemission spectroscopy," Phys. Rev. B **84**, 064519 (2011).
